# Chlorpromazine and Amitriptyline Are Substrates and Inhibitors of the AcrB Multidrug Efflux Pump

**DOI:** 10.1128/mBio.00465-20

**Published:** 2020-06-02

**Authors:** Elizabeth M. Grimsey, Chiara Fais, Robert L. Marshall, Vito Ricci, Maria Laura Ciusa, Jack W. Stone, Alasdair Ivens, Giuliano Malloci, Paolo Ruggerone, Attilio V. Vargiu, Laura J. V. Piddock

**Affiliations:** a Antimicrobials Research Group, School of Immunity and Infection, College of Medical and Dental Sciences, Institute of Microbiology and Infection, The University of Birmingham, Birmingham, United Kingdom; b Department of Physics, University of Cagliari, Monserrato, Italy; c Centre for Immunity, Infection and Evolution, University of Edinburgh Ashworth Labs, Edinburgh, United Kingdom; d Bijvoet Center for Biomolecular Research, Faculty of Science—Chemistry, Utrecht University, Utrecht, The Netherlands; University of British Columbia

**Keywords:** antibiotic resistance, efflux pump inhibitors, AcrB, antipsychotic drugs, efflux pumps

## Abstract

Efflux is an important mechanism in Gram-negative bacteria conferring multidrug resistance. Inhibition of efflux is an encouraging strategy to restore the antibacterial activity of antibiotics. Chlorpromazine and amitriptyline have been shown to behave as efflux inhibitors. However, their mode of action is poorly understood. Exposure of Salmonella enterica serovar Typhimurium and Escherichia coli to chlorpromazine selected for mutations within genes encoding RamR and MarR, regulators of the multidrug tripartite efflux pump AcrAB-TolC. Further experiments with *S.* Typhimurium containing AcrB D408A (a nonfunctional efflux pump) and chlorpromazine or amitriptyline resulted in the reversion of the mutant *acrB* allele to the wild type. Together, this suggests these drugs are AcrB efflux substrates. Subsequent docking studies with AcrB from *S.* Typhimurium and E. coli, followed by molecular dynamics simulations and free energy calculations showed that chlorpromazine and amitriptyline bind at the hydrophobic trap, a preferred binding site for substrates and inhibitors within the distal binding pocket of AcrB. Based on these simulations, we suggest that chlorpromazine and amitriptyline inhibit AcrB-mediated efflux by interfering with substrate binding. Our findings provide evidence that these drugs are substrates and inhibitors of AcrB, yielding molecular details of their mechanism of action and informing drug discovery of new efflux inhibitors.

## Abstract (precis)

Efflux pumps of the resistance nodulation-cell division (RND) superfamily are major contributors to multidrug resistance for most of the Gram-negative ESKAPE (Enterococcus faecium, Staphylococcus aureus, Klebsiella pneumoniae, Acinetobacter baumannii, Pseudomonas aeruginosa, and *Enterobacter* species) pathogens. The development of inhibitors of these pumps would be highly desirable; however, several issues have thus far hindered all efforts at designing new efflux inhibitory compounds devoid of adverse effects. An alternative route to *de novo* design relies on the use of marketed drugs, for which side effects on human health have been already assessed. In this work, we provide experimental evidence that the antipsychotic drugs chlorpromazine and amitriptyline are inhibitors of the AcrB transporter, the engine of the major RND efflux pumps in Escherichia coli and Salmonella enterica serovar Typhimurium. Furthermore, *in silico* calculations have provided a molecular-level picture of the inhibition mechanism, allowing rationalization of experimental data and paving the way for similar studies with other classes of marketed compounds.

## INTRODUCTION

Multidrug resistance (MDR) efflux pumps constitute one of the most prevalent intrinsic drug resistance mechanisms that are universally conserved across bacterial species (1). Efflux pumps regulate the intracellular environment by extruding toxic substrates, including secondary metabolites, quorum sensing molecules, dyes, biocides, and antibiotics (2). Drug resistance results from the active expulsion of a given drug causing a reduction in the intracellular concentration and thus antimicrobial potency (3). In Gram-negative bacteria, the major MDR efflux pumps belong to the resistance nodulation-cell division (RND) family of transporters, which form systems that span the entire cell envelope (2). The AcrAB-TolC complex found in *Enterobacterales* is a model efflux system. AcrAB-TolC forms a tripartite complex consisting of an inner membrane pump protein (AcrB) and an outer membrane channel protein (TolC) bridged by a periplasmic adaptor protein (AcrA) (2). AcrB is a homotrimeric secondary antiporter with a jellyfish-like structure that has been crystallized in both the putative resting symmetric state (4) and asymmetric conformations (5, 6). In the latter arrangement, each monomer can assume a different structure (loose [L], tight [T], or open [O]) corresponding to a different functional state in relation to substrate export, which is believed to occur through a functional rotation mechanism involving peristaltic motions of internal protein channels (7, 8). AcrB utilizes the proton motive force as an energy source to drive export of a wide range of structurally diverse substrates against their concentration gradient (9). Some substrates have been cocrystallized while bound at different locations of the protein, either on its surface (10), at peripheral binding sites such as the so-called access pocket on the L protomer (11, 12), or at more buried pockets such as the distal pocket on the T protomer (DP_T_) (5, 12, 13). Deletion or inactivation of any gene encoding a component of this efflux pump confers hypersusceptibility to pump substrates (14–18). Unlike deletion mutants, point mutants that ablate the function of AcrB (without loss of protein) do not result in overexpression of other RND efflux pumps (17). This suggests that not only are gene deletion mutants unsuitable for the study of membrane transporters but also that inhibitors of AcrB may not cause increased expression of alternative RND pumps.

Considering their role in innate and evolved resistance, efflux pumps are targets for the discovery and development of antimicrobial adjuvants (19); their inhibition prevents the extrusion of antibiotics to restore their antibacterial activity (20–24). Currently identified efflux inhibitor classes include peptidomimetics (25), piperazines (26), pyridopyrimidines (27), and most recently, the pyranopyrimidines (28). However, none have been approved for clinical use as efflux inhibitors largely due to their cytotoxicity (29).

One strategy to identify potential efflux inhibitors is to screen and repurpose drugs already in clinical use for indications other than infectious diseases (29). Considering their pharmacokinetics and toxicology are well described, their use may be invaluable in terms of bypassing the time and costs associated with drug development. Among the drugs considered for repurposing, there is evidence that the first-generation antipsychotic medications chlorpromazine and amitriptyline behave as efflux inhibitors (30, 31). Chlorpromazine has also been shown to possess antimicrobial activities (30, 32, 33). While these activities occur at concentrations greater than those clinically achievable and/or desirable, chlorpromazine is able to potentiate the activities of many antibiotics at subinhibitory concentrations (30, 34–36) and increase the accumulation of ethidium bromide and other AcrB substrates (30, 37, 38). However, the mechanism by which this occurs is unknown. Less is known about the efflux inhibitory effects of amitriptyline. However, like chlorpromazine, amitriptyline potentiates antibiotic activity; hypersusceptibility to amitriptyline occurs when *ramA* is deleted in Salmonella enterica serovar Typhimurium, and exposure to amitriptyline results in the induction of *ramA* (30). The latter has been previously associated with lack of efflux (39).

Mechanistic studies regarding the interaction between RND transporters and their substrates/inhibitors (see reference 8 for a recent review) have provided useful insights into the molecular determinants of polyspecificity (8, 40–44), the mechanisms of active transport of substrates (8, 45–50), and the putative inhibition or modulation of transport routes (20, 22, 51–54). In particular, studies performed by the authors identified key structural determinants discriminating between substrates and inhibitors of AcrB in Escherichia coli (41), which were later confirmed by experimental findings (22).

Here, exposure to growth-inhibitory concentrations of chlorpromazine resulted in the selection of mutants containing mutations within genes encoding RamR and MarR, regulators of AcrAB-TolC, in *S.* Typhimurium and E. coli, respectively. Further mutant selection experiments with *S.* Typhimurium containing a nonfunctional efflux pump (AcrB D408A), chlorpromazine, and amitriptyline reverted the mutant to the wild-type *acrB* allele. Together, these data suggest that these drugs are AcrB efflux substrates. This hypothesis was corroborated by multiple *in silico* investigations of the interaction of both compounds with AcrB of *S.* Typhimurium and E. coli. Given their ability to bind AcrB, we suggest that chlorpromazine and amitriptyline are able to exert their experimentally observed efflux inhibition by interfering with the binding of other AcrB substrates.

## RESULTS

### Exposure to chlorpromazine selects for mutations in AcrAB-TolC regulatory genes.

The MIC of chlorpromazine for E. coli MG1655 and *S.* Typhimurium SL1344 was 256 μg/ml. Until recently, it was thought that selection of antibiotic-resistant bacteria only occurs in the “mutant-selective window,” i.e., the range of antibiotic concentrations between the MIC of the susceptible population and that of the resistant population. Therefore, mutants were initially selected using concentrations one and two times the MIC of the parental strain. However, at these concentrations, no resistant mutants were obtained. Andersson et al. revealed that very low concentrations of compound can select for resistance in both laboratory and natural environments (55, 56). Therefore, sub-MICs of chlorpromazine (170 μg/ml and 150 μg/ml) were used to select for E. coli and *S.* Typhimurium mutants, respectively. Low mutation rates of 4.95 × 10^−13^ CFU/ml (E. coli) and 1.16 × 10^−10^ CFU/ml (*S.* Typhimurium) were calculated. Each mutant was subjected to MIC testing using a panel of antibiotics that represent a variety of classes. This MIC determination revealed that each mutant was 1-to 2-fold less susceptible to chlorpromazine than the parental strain (512 μg/ml to 1,024 μg/ml). Usually, a 1-fold difference in an MIC value is not considered to be signiﬁcant, as this can lie within the error of the method. However, given that this change in MIC was repeatedly observed, these mutants were considered to have decreased susceptibility to chlorpromazine.

Each mutant was grouped depending on its chlorpromazine MIC phenotype, and one representative clone from each group was sent for whole-genome sequencing (WGS). The analysis revealed mutations in coding regions of the genomes of each mutant. A single nucleotide polymorphism (SNP) resulting in a nonsynonymous mutation (L158P) in *ramR* was observed for the single *S.* Typhimurium mutant. The two E. coli mutants contained deletions in *marR*: 141_142del and 104delC. RamR and MarR are transcriptional regulators that repress expression of the AcrAB-TolC efﬂux pump.

### The mutated gene conferring AcrB D408A in *S.* Typhimurium reverts to the wild-type allele upon exposure to chlorpromazine and amitriptyline.

Mutant selection experiments using *S.* Typhimurium SL1344 containing a substitution (D408A) in AcrB rendering it nonfunctional were performed to determine the impact of chlorpromazine on a strain lacking a functional AcrAB-TolC MDR efflux pump (Table 1). In total, 152 mutants were selected upon exposure to chlorpromazine and were determined by MIC to be resistant to the selecting agent; the mutation frequency and rate were 1.82 × 10^−10^ and 5.94 × 10^−10^ mutations per cell/per generation, respectively. Quantitative PCR of 100 mutants revealed that 100% of the mutants had reverted from the mutant allele (1223G) to the wild-type sequence (1223T). Subsequently, this experiment was repeated with amitriptyline. Given that it has been suggested that amitriptyline possesses efflux inhibitory properties, it was included to determine whether reversion occurs upon exposure to chlorpromazine only or is a feature of other tricyclic drugs capable of efflux inhibition. Consistent with chlorpromazine, of the 43 mutants selected upon exposure to amitriptyline (mutation frequency and rate of 3.87 × 10^−11^ and 1.44 × 10^−10^ mutations per cell/per generation, respectively), in 100% of the mutants, the *acrB* sequence reverted to the wild-type sequence. We hypothesized that there would be no evolutionary benefit to this reversion if these compounds were not AcrB substrates and, further, that these data indicate that chlorpromazine and amitriptyline are substrates of AcrB. To test our hypothesis, this experiment was repeated with the known AcrB substrates minocycline and ethidium bromide and, as a control, to a nonsubstrate, spectinomycin, to determine if this is a feature common among AcrB substrates or a feature unique to compounds with efflux inhibitory properties.

**TABLE 1:** Frequency and rate of mutation and reversion rate of the D408A mutation when *S*. Typhimurium AcrB (D408A) was exposed to chlorpromazine, amitriptyline, minocycline, spectinomycin, and ethidium bromide

Selecting drug	Selecting concn (μg/ml)	Mutation frequency (CFU/ml)	Mutation rate (CFU/ml)	Total no. of mutants	Reversion rate (%)
Chlorpromazine	60	1.82 × 10^−10^	5.94 × 10^−10^	152	100
Amitriptyline	110	3.87 × 10^−11^	1.44 × 10^−10^	43	100
Minocycline	0.5	6.48 × 10^−10^	1.399 × 10^−9^	702	2
Ethidium bromide	64	4.04 × 10^−9^	4.95 × 10^−9^	4,199	3
Spectinomycin	128	5.46 × 10^−10^	1.34 × 10^−9^	459	0

In total, 702, 4,199, and 459 mutants were selected with minocycline (mutation frequency and rate of 6.48 × 10^−10^ and 1.399 × 10^−9^ mutations per cell/per generation, respectively), ethidium bromide (mutation frequency and rate of 4.04 × 10^−9^ and 4.95 × 10^−9^ mutations per cell/per generation, respectively), and spectinomycin, respectively (mutation frequency and rate of 5.46 × 10^−10^ and 1.34 × 10^−9^ mutations per cell/per generation, respectively). Quantitative PCR of 100 mutants revealed that only 2% of the minocycline-resistant mutants and 3% of the ethidium bromide-resistant mutants reverted to the wild-type allele. In contrast, none of the mutants selected upon exposure to spectinomycin had reverted.

### RNA sequencing reveals upregulation of transcriptional regulators of AcrAB-TolC.

**FIG 1:**
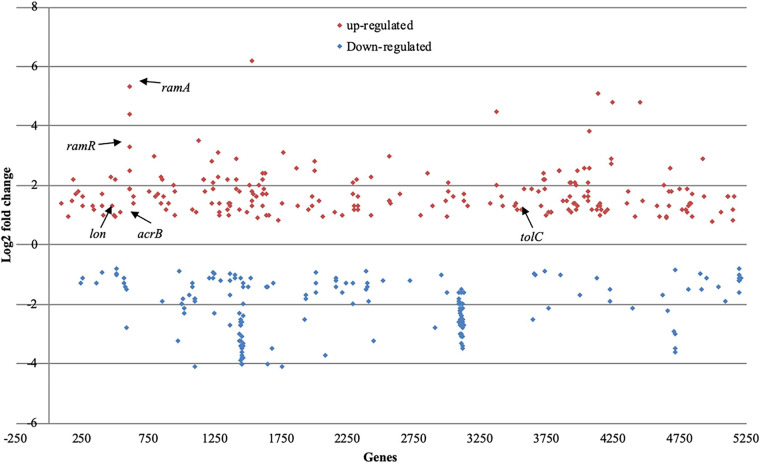
Transcriptional profile of *S.* Typhimurium SL1344 after exposure to 50 μg/ml of chlorpromazine in comparison to that of unexposed SL1344. Significantly upregulated genes are shown in red, while downregulated genes are shown in blue.

To understand the physiological effects that occur upon exposure to 50 μg/ml of chlorpromazine, changes in the gene expression of *S.* Typhimurium SL1344 60 min after exposure to this compound were determined by sequencing isolated RNA. Relative to those in the unexposed control strain, in the presence of chlorpromazine, 6.5% of the genes in SL1344 were differentially transcribed (Fig. 1). Of these differentially transcribed genes, *acrB*, *tolC*, *ramA*, *lon*, and *ramR* were upregulated. Each of these genes are involved in the expression and regulation of the AcrAB-TolC multidrug efflux pump.

**FIG 2:**
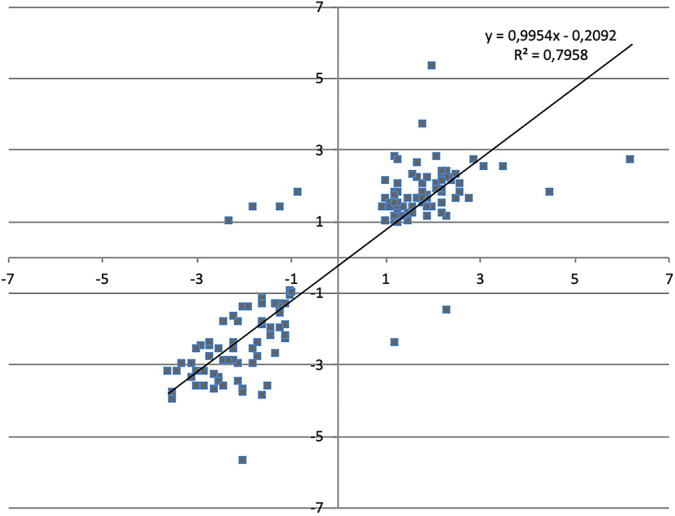
Comparison of the Log fold change (LogFC) values between *S.* Typhimurium SL1344 exposed to chlorpromazine or PaβN for each significantly transcribed gene. A Pearson’s correlation was used to determine the *R*^2^ value.

**FIG 3:**
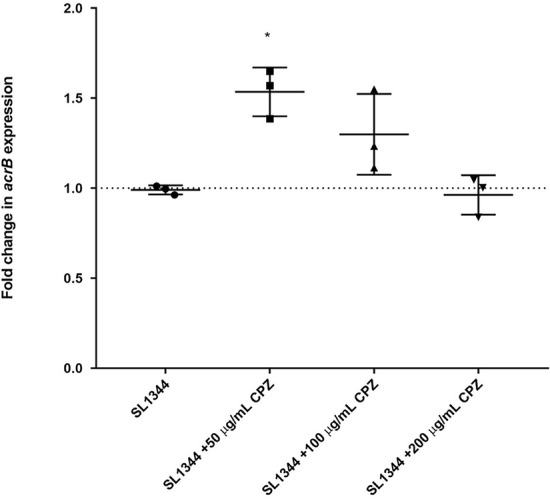
Fold change in normalized *acrB* expression in SL1344 ± chlorpromazine at 50, 100, and 200 μg/ml. Data were analyzed by a Student's *t* test with Welch’s correction. *, *P* < 0.05 versus the untreated control.

We then compared the transcriptome of SL1344 exposed to chlorpromazine against that of SL1344 exposed to the known efflux pump inhibitor Phe-Arg-β-naphthylamide (PaβN) (Fig. 2). In total, there were 147 genes that were significantly changed under both conditions. Of these, 141 (95.92%) were changed in the same direction, suggesting that chlorpromazine behaves in a similar manner to PaβN. A positive correlation between the two data sets is quantified by an *R*^2^ value of 0.79. The paired-end RNA sequencing (RNA-seq) data for both chlorpromazine and PaβN are available in ArrayExpress (accession no. E-MTAB-8190). Previously, Bailey et al. reported that upon exposure to 200 μg/ml of chlorpromazine, the expression of *acrB* was repressed by 40% in relation to that in the unexposed wild type, despite an increase in expression of the transcriptional activator *ramA* (30). Here, we also saw a statistically nonsignificant reduction in the expression of *acrB* at 200 μg/ml. However, in support of the RNA sequencing at 50 μg/ml and 100 μg/ml (concentrations at which efflux inhibition is observed), we saw an increase in the expression of *acrB* by 1.54- (54%) and 1.30-fold (30%) (Fig. 3).

### Chlorpromazine and amitriptyline are able to potentiate the activity of AcrB substrates in a species-dependent manner.

Both chlorpromazine and amitriptyline have some intrinsic antibacterial activity. Potentiation assays were performed in combination with a range of substrates of the AcrAB-TolC efflux pump: chloramphenicol, nalidixic acid, tetracycline, ciprofloxacin, norfloxacin, and ethidium bromide against E. coli and *S.* Typhimurium (both possessing the RND efflux pump AcrAB-TolC) as well as Pseudomonas aeruginosa and Acinetobacter baumannii (possessing the RND efflux pumps MexAB-OprM and AdeABC, respectively). A range of substrates was used to determine whether chlorpromazine and amitriptyline selectively potentiate the activity of a single antibiotic/class or are broad spectrum. In addition, the use of several Gram-negative species, of which, A. baumannii and P. aeruginosa do not possess AcrAB-TolC, provides information on whether chlorpromazine and amitriptyline are effective against AcrAB-TolC alone or are able to target homologous efflux pumps. Strains overexpressing efflux pumps were used because the strain will efflux to a greater extent than the parental wild-type strain; thus, larger changes in efflux activity will be observed in the presence of chlorpromazine and amitriptyline.

In combination with antibiotics, amitriptyline increased the susceptibility of *S.* Typhimurium overexpressing AcrAB-TolC to chloramphenicol, nalidixic acid, and tetracycline but not to ciprofloxacin or ethidium bromide (Table 2). Amitriptyline also increased the susceptibility of A. baumannii to chloramphenicol, while in P. aeruginosa, the only antibiotic with potentiated activity was nalidixic acid. Chlorpromazine increased the susceptibility of *S.* Typhimurium to chloramphenicol, ciprofloxacin, tetracycline, and nalidixic acid but not to ethidium bromide (Table 3). Chlorpromazine increased the susceptibility of P. aeruginosa to chloramphenicol, nalidixic acid, tetracycline, and ethidium bromide but not to ciprofloxacin. Chlorpromazine did not increase the susceptibility of A. baumannii to any of the tested substrates.

**TABLE 2:** MIC of various compounds against *Salmonella* Typhimurium SL1344 *ramR*::*aph*, Escherichia coli BW25113 *marR*::*aph*, Acinetobacter baumannii AB211, and Pseudomonas aeruginosa PAO1 exposed to various compounds alone and in combination with amitriptyline

Antibiotics	Amitriptyline concn (fraction of MIC)	MIC (μg/ml)^*a*^			
		SL1344 *ramR*::*aph*	BW25113 *marR*::*aph*	AB211	PAO1
Chloramphenicol	None	16	4	256	256
	^1^/_16_	8	4	256	128
	^1^/_8_	**4**	4	128	128
	^1^/_4_	**2**	2	**64**	128
Ciprofloxacin	None	0.06	0.004	512	0.25
	^1^/_16_	0.06	0.03	512	0.25
	^1^/_8_	0.03	0.008	512	0.25
	^1^/_4_	0.03	0.004	512	0.125
Nalidixic acid	None	8	4	1,024	1,024
	^1^/_16_	8	4	512	512
	^1^/_8_	4	4	512	**256**
	^1^/_4_	**2**	2	512	**256**
Tetracycline	None	8	2	256	64
	^1^/_16_	4	2	256	64
	^1^/_8_	**2**	2	256	64
	^1^/_4_	**1**	1	256	32
Norfloxacin	None	0.25	0.03	512	1
	^1^/_16_	0.12	0.06	512	1
	^1^/_8_	0.25	0.03	512	1
	^1^/_4_	0.25	0.03	512	1
Ethidium bromide	None	512	128	256	2,048
	^1^/_16_	512	64	256	2,048
	^1^/_8_	512	128	256	2,048
	^1^/_4_	256	64	128	1,024

a The MIC of amitriptyline was 888 μg/ml, 444 μg/ml, 222 μg/ml, and 1,775 μg/ml against SL1344 *ramR::aph*, BW25113 *marR::aph*, AB211, and PAO1, respectively. Bold font indicates a ≥2-fold decrease in MIC value in comparison to the antibiotic alone.

**TABLE 3:** MIC of various compounds against *Salmonella* Typhimurium SL1344 *ramR*::*aph*, Escherichia coli BW25113 *marR*::*aph*, Acinetobacter baumannii AB211, and Pseudomonas aeruginosa PAO1 exposed to various compounds alone and in combination with chlorpromazine

Antibiotics	Chlorpromazine concn (fraction of MIC)	MIC (μg/ml)^*a*^			
		SL1344 *ramR*::*aph*	BW25113 *marR*::*aph*	AB211	PAO1
Chloramphenicol	None	8	4	64	128
	^1^/_16_	4	2	64	64
	^1^/_8_	**2**	2	64	**32**
	^1^/_4_	**1**	2	32	**32**
Ciprofloxacin	None	0.03	0.008	128	0.25
	^1^/_16_	0.03	0.008	128	0.25
	^1^/_8_	0.015	0.008	128	0.25
	^1^/_4_	**0.008**	0.004	128	0.25
Nalidixic acid	None	8	4	512	512
	^1^/_16_	4	2	512	**128**
	^1^/_8_	4	2	512	**128**
	^1^/_4_	**0.5**	2	512	**64**
Tetracycline	None	4	2	512	32
	^1^/_16_	4	1	512	16
	^1^/_8_	2	1	256	**8**
	^1^/_4_	**1**	1	256	**8**
Norfloxacin	None	0.25	0.03	256	1
	^1^/_16_	0.25	0.03	512	1
	^1^/_8_	0.25	0.06	512	1
	^1^/_4_	0.5	0.06	256	1
Ethidium bromide	None	1,024	128	128	4,096
	^1^/_16_	512	128	64	**1,024**
	^1^/_8_	512	64	64	**1,024**
	^1^/_4_	512	64	64	**256**

a The MIC of chlorpromazine was 1,024 μg/ml, 256 μg/ml, 128 μg/ml, and 2,048 μg/ml against SL1344 *ramR*::*aph*, BW25113 *marR*::*aph*, AB211, and PAO1, respectively. Bold font indicates a ≥2-fold decrease in MIC value in comparison to the antibiotic alone.

**FIG 4:**
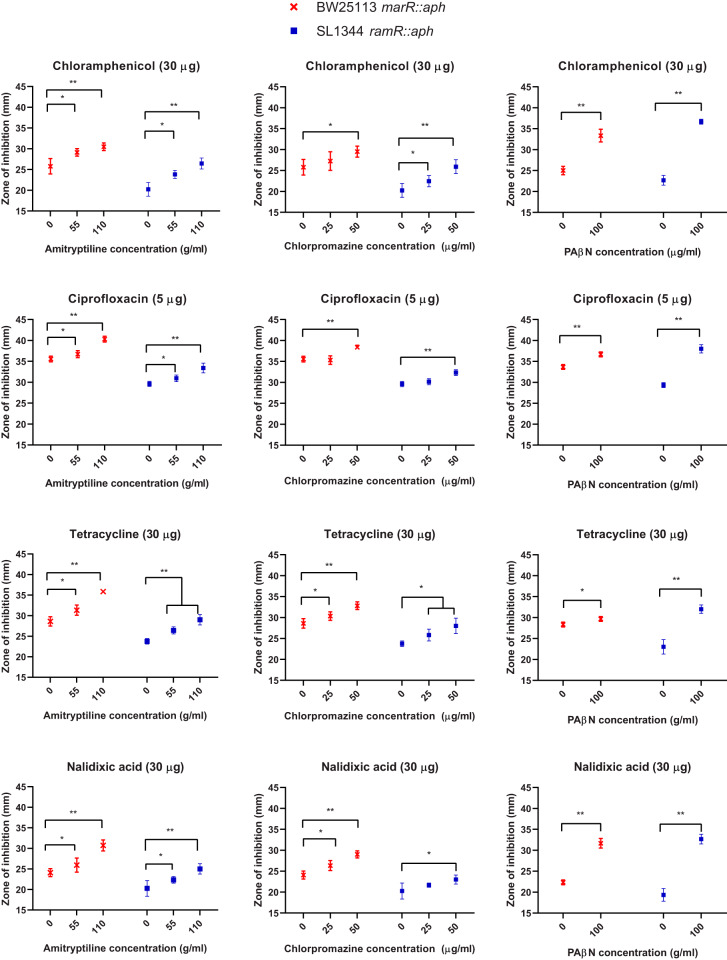
Comparisons of the zones of inhibition obtained for disks containing chloramphenicol, ciprofloxacin, amitriptyline, tetracycline, and nalidixic acid when used in combination with chlorpromazine, amitriptyline, and the positive-control PaβN. Data were analyzed by a Student's *t* test with Welch’s correction. *, *P* < 0.05; **, *P* < 0.001 versus the untreated control.

**FIG 5:**
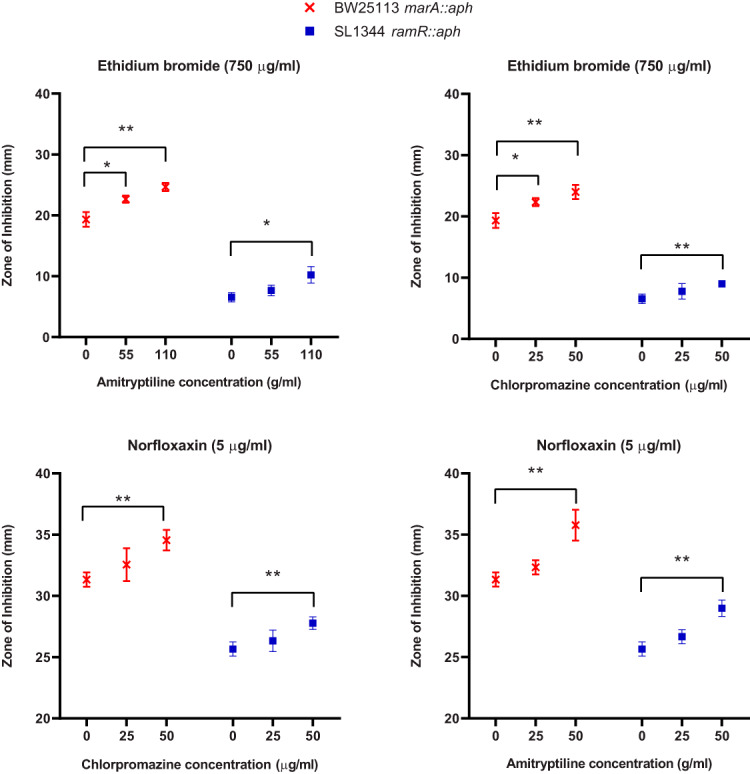
Comparisons of the zones of inhibition obtained for well diffusion assay with ethidium bromide and norfloxacin when used in combination with chlorpromazine and amitriptyline. Data were analyzed by a Student's *t* test with Welch’s correction. *, *P* < 0.05; **, *P* < 0.001 versus the untreated control.

Interestingly, when tested in a checkerboard assay, neither chlorpromazine nor amitriptyline potentiated the activity of norfloxacin against any strain or of any of the tested substrates against E. coli overexpressing AcrAB-TolC. Therefore, a disk diffusion assay in which Iso-Sensitest agar plates were supplemented with various concentrations of chlorpromazine and amitriptyline was used to assess the effect of these compounds on the antibiotic susceptibility of E. coli and *S.* Typhimurium that overexpressed AcrAB-TolC. In addition, the known efflux inhibitor PaβN was used as a positive control. For both E. coli and *S.* Typhimurium, the zones of inhibition for chloramphenicol, tetracycline, ciprofloxacin, and nalidixic acid were significantly larger in the presence of chlorpromazine, amitriptyline, and PaβN than in their absence (Fig. 4). Finally, due to the nature of ethidium bromide and the high potency of norfloxacin when used in disk form against these strains, a disk diffusion assay could not be performed. Thus, a well diffusion assay was undertaken in which ethidium bromide-norfloxacin was incorporated into holes within Iso-Sensitest agar plates supplemented with increasing concentrations of chlorpromazine and amitriptyline. For both E. coli BW25113 *marR*::*aph* and *S.* Typhimurium SL1344 *ramR*::*aph*, the zone of inhibition for ethidium bromide and norfloxacin was significantly larger in the presence of amitriptyline and chlorpromazine than in their absence (Fig. 5).

### Chlorpromazine and amitriptyline exhibit efflux inhibitory effects.

**FIG 6:**
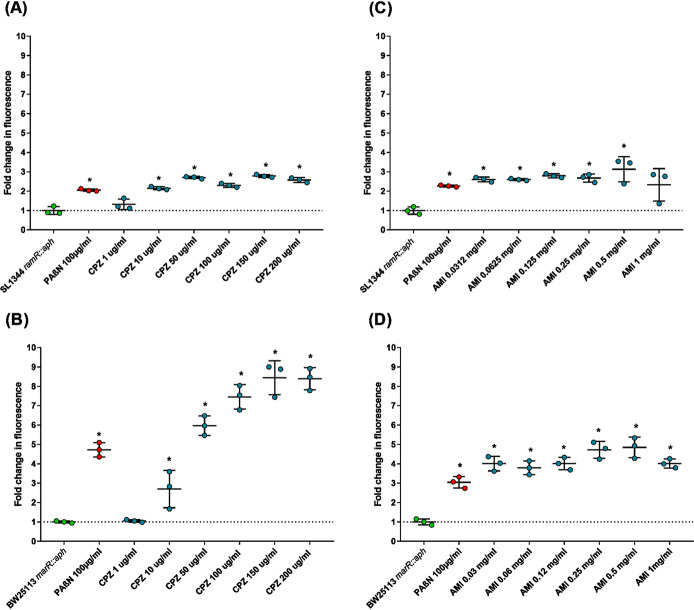
Efflux of ethidium bromide in the presence of chlorpromazine or amitriptyline. Efflux of ethidium bromide in the presence of chlorpromazine in *S.* Typhimurium SL1344 *ramR*::*aph* (A) and E. coli BW25113 *marR*::*aph* (B). Efflux of ethidium bromide in the presence of amitriptyline in *S.* Typhimurium SL1344 *ramR*::*aph* (C) and E. coli BW25113 *marR*::*aph* (D). Data were analyzed by a Student's *t* test with Welch’s correction. *, *P* < 0.05 versus the untreated control; CPZ, chlorpromazine; AMI, amitriptyline.

The efflux activity of the AcrAB-TolC-overexpressing strains *S.* Typhimurium SL1344 *ramR*::*aph* and E. coli BW25113 *marR*::*aph* was measured by determining the efflux of ethidium bromide and the intracellular accumulation of norfloxacin; a known efflux inhibitor, PaβN, was used as a positive control. Unfortunately, due to limited fluorescence, efflux and accumulation assays could not be performed with all substrates used in the synergy assays. Therefore, well-described substrates of AcrB, ethidium bromide and norfloxacin, which are fluorescent either intrinsically (norfloxacin) or upon intercalation with DNA (ethidium bromide), were used for the phenotypic assays and in the subsequent *in silico* models (57, 58). Compared with that in untreated controls, increasing concentrations of chlorpromazine significantly decreased the efflux of ethidium bromide by E. coli and *S.* Typhimurium (Fig. 6). Interestingly, all concentrations of amitriptyline decreased the efflux of ethidium bromide to a similar extent, suggesting that this compound saturates the pump at relatively low concentrations. At the highest concentrations of chlorpromazine and amitriptyline, the decrease in ethidium bromide efflux was comparable to, or greater than, the inhibition by PaβN. A greater decrease in ethidium bromide efflux was seen for E. coli, in which the largest decrease in efflux was 8.44-fold in the presence of 150 μg/ml of chlorpromazine and 4.84-fold in the presence of 0.5 mg/ml of amitriptyline. In comparison, 2.78- and 3.12-fold changes were observed for *S.* Typhimurium at the same concentrations of chlorpromazine and amitriptyline, respectively. Importantly, previous studies have shown that the ability of chlorpromazine to decrease efflux of ethidium bromide is ablated in strains lacking AcrB, suggesting that its efflux inhibitory activity is specific to AcrB (30). In addition, both chlorpromazine and amitriptyline significantly decreased the efflux of ethidium bromide and Hoechst H33342 from wild-type E. coli BW25113 and *S.* Typhimurium SL1344 (data not shown).

**FIG 7:**
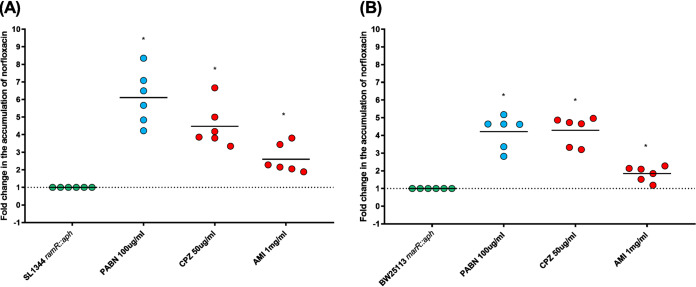
Accumulation of norfloxacin in the presence of chlorpromazine and amitriptyline. (A) Fold change in accumulation of norfloxacin in the presence of chlorpromazine and amitriptyline in *S.* Typhimurium SL1344 *ramR*::*aph.* (B) Fold change in accumulation of norfloxacin upon exposure to chlorpromazine and amitriptyline in E. coli BW25113 *marR*::*aph.* Data were analyzed by a Student's *t* test with Welch’s correction. *, *P* < 0.05 versus the untreated control; CPZ, chlorpromazine; AMI, amitriptyline.

Statistically significant increases in the intracellular concentration of norfloxacin in the presence of chlorpromazine (50 μg/ml) or amitriptyline (1 mg/ml) (4.50- and 4.28-fold for chlorpromazine, and 2.50 and 1.83-fold for amitriptyline) were observed with the AcrAB-TolC-overexpressing strains *S.* Typhimurium SL1344 *ramR*::*aph* and E. coli BW25113 *marR*::*aph*, respectively (Fig. 7).

### Amitriptyline and chlorpromazine bind to the same region of AcrB as known substrates and inhibitors.

To investigate if the inhibitory action of chlorpromazine and amitriptyline could be rationalized in terms of their interaction with AcrB, their propensity to bind to this transporter in both E. coli and *S.* Typhimurium (here referred to as AcrB_EC_ and AcrB_ST_, respectively) was assessed by means of docking calculations, molecular dynamics (MD) simulations and free energy estimations.

The blind ensemble docking campaign performed for both chlorpromazine and amitriptyline resulted in up to 200 poses per ligand. The overall distributions of their putative binding poses overlapped fairly well in both AcrB_EC_ and AcrB_ST_ (see Fig. S1 in the supplemental material). Importantly, all distributions featured a large number of high-affinity poses within the DP_T_ and in tight interaction with the so-called hydrophobic trap (HT; lined by phenylalanine residues F136, F178, F610, F615, and F628 in both AcrB_EC_ and AcrB_ST_) (see Table S1). This site is known to be a preferred binding region for inhibitors such as PAβN (20, 54), 1-(1-naphtylmethyl)-piperazine (NMP) (41), D13-9001 (59), and the MBX compound series (22, 54). In addition, chlorpromazine and amitriptyline displayed, on average, similar docking scoring energies on AcrB_EC_ and AcrB_ST_ (Table 4).

**TABLE 4:** (Pseudo)binding free energies evaluated through the scoring function of AutoDock VINA for the top ranked poses of both amitriptyline and chlorpromazine on AcrB_EC_ and AcrB_ST_

Complex^*a*^	Δ*G*_max_ (kcal/mol)
AMI-AcrB_EC_	−11.6
AMI-AcrB_ST_	−12.1
CPZ-AcrB_EC_	−9.2
CPZ-AcrB_ST_	−9.3

a All corresponding poses are localized within the DP_T_. CPZ, chlorpromazine; AMI, amitriptyline.

10.1128/mBio.00465-20.1 FIG S1 Distribution of the top docking poses obtained from blind ensemble docking calculations of chlorpromazine (CPZ), amitriptyline (AMI), norfloxacin (NOR), and ethidium bromide (EtBr) on AcrB_EC_ (A) and on AcrB_ST_ (B) (see Materials and Methods for details). The picture shows the distribution of the centers of mass of the poses, colored according to scoring function of AutoDock VINA (Δ*G*_pseudo_). The protein is shown in transparent ribbons, with monomers L and T in the front, on the left and right side of the central intermonomer vestibule. The transparency increases going from monomer T to L to O. The sidechains of phenylalanines lining the HT of monomer T are shown as magenta sticks. Download FIG S1, PDF file, 0.3 MB. Copyright © 2020 Grimsey et al. 2020 Grimsey et al. This content is distributed under the terms of the Creative Commons Attribution 4.0 International license.

10.1128/mBio.00465-20.6 TABLE S1 Residues lining the regions of interest of AcrB. The same definitions can be used for AcrB_EC_ and AcrB_ST_, due to the lack of gaps between their sequences. Download Table S1, DOCX file, 0.01 MB. Copyright © 2020 Grimsey et al. 2020 Grimsey et al. This content is distributed under the terms of the Creative Commons Attribution 4.0 International license.

To provide molecular-level insights on the possible mechanism by which chlorpromazine and amitriptyline interfere with the efflux of ethidium bromide and alter the intracellular accumulation of norfloxacin, we also performed blind ensemble docking calculations of norfloxacin and ethidium bromide on both AcrB_EC_ and AcrB_ST_. Importantly, the distributions of preferred putative binding sites of these AcrB substrates significantly overlapped those obtained for chlorpromazine and amitriptyline. Moreover, most of the highest affinity poses were localized within the DP_T_ (Fig. S1).

To investigate in more detail the structural and dynamic features as well as the thermodynamics of binding of all compounds within the DP_T_, we carried out multiple all-atom MD simulations and binding free energy calculations. According to previous work (60, 61), we performed a cluster analysis of the binding poses within the DP_T_. This resulted in three clusters grouping together in almost one-half of all docking conformations for amitriptyline and chlorpromazine within the DP_T_ of AcrB_EC_ and AcrB_ST_, while for ethidium bromide and norfloxacin, the same coverage was achieved with one cluster only. Thus, the representatives of clusters 1 to 3 (sorted by population) were used as starting structures for MD simulations for amitriptyline and chlorpromazine. For ethidium bromide and norfloxacin, three different structures from the first cluster were chosen as starting structures for MD simulations. A total of 24 all-atom MD simulations (see Table S2), each having a production run of 150 ns in length, were performed using a truncated model of AcrB that was validated in previous works (20, 41, 54) (see Materials and Methods).

10.1128/mBio.00465-20.7 TABLE S2 MD simulations performed in this work. The starting configurations of each substrate were selected among the clusters of the docking poses localized within the DP_T_. CPZ, chlorpromazine; AMI, amitriptyline. Download Table S2, PDF file, 0.2 MB. Copyright © 2020 Grimsey et al. 2020 Grimsey et al. This content is distributed under the terms of the Creative Commons Attribution 4.0 International license.

**FIG 8:**
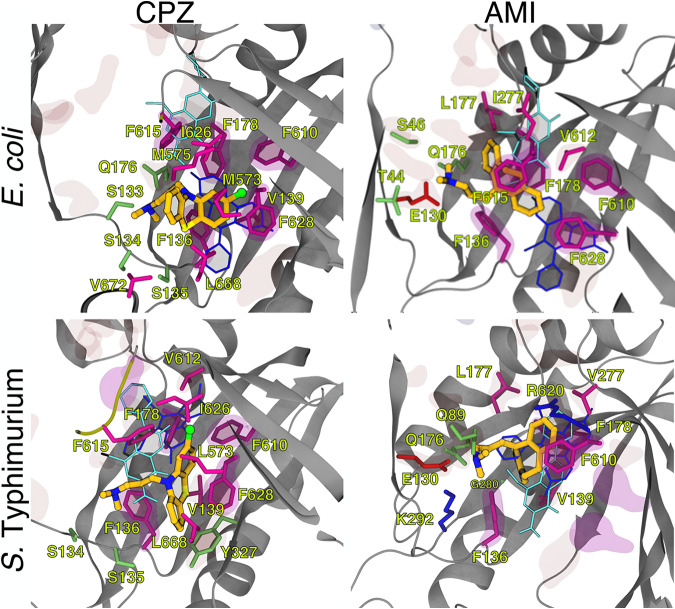
Representative conformations of the most stable binding modes of chlorpromazine and amitriptyline within the DP_T_ of AcrB_EC_ and AcrB_ST_, as obtained from all-atom MD simulations of the periplasmic portion of the transporter in explicit solvent (see Materials and Methods for details and Fig. S3 in the supplemental material). The protein is shown as gray ribbons, the inhibitors as CPK colored by element (C, N, S, and Cl in dark yellow, blue, light yellow, and green, respectively). Side chains of residues within 3.5 Å of the inhibitors are also shown as sticks colored by residue type (hydrophobic, polar, acid, and basic in purple, lime, red, and blue, respectively) and labeled. Side chains of residues defining the DP_T_ and the phenylalanines lining the HT (see Table S1 for the definition of different protein regions) are also shown in transparent red and magenta surfaces, respectively. The most stable conformations of norfloxacin and ethidium bromide as obtained also from all-atom MD simulations are shown for comparison in cyan and blue sticks, respectively.

Importantly, inspection of MD trajectories revealed that all compounds bound stably to the DP_T_ region (Fig. 8 and S2). For the sake of clarity, in the following, we discuss only the results obtained for the most stable trajectory of each compound (see Fig. S3). In their stable conformations, both chlorpromazine and amitriptyline were partly embedded within the HT of the DP_T_, making 10 (chlorpromazine-AcrB_EC_ and chlorpromazine-AcrB_ST_), 8 (amitriptyline-AcrB_EC_), and 6 (amitriptyline-AcrB_ST_) direct contacts with hydrophobic residues within this pocket (a contact was counted when the minimum ligand-residue distance was less than 3.5 Å) (Fig. 8). Notably, chlorpromazine occupies a significant fraction of the pocket where well-known AcrB inhibitors, such as MBX3132 in AcrB_EC_ (22), were shown to bind. Consistently, chlorpromazine features a larger steric clash than amitriptyline with MBX3132 upon superimposition of their complex structures with the MBX3132-AcrB_EC_ crystallographic structure (Table 5). Compared to chlorpromazine, amitriptyline binds somewhat upside the DP_T_, making direct hydrophilic contacts with residues E130 and Q176 through its dimethylamine group, as well as occasional water-mediated interactions with AcrB (Fig. 8 and Table S3). The same amine is involved in direct cation-π, H-bonding, and water-mediated interactions between chlorpromazine and AcrB. The different interactions made by the two compounds are mirrored in the contribution of the residues lining the DP_T_ (and the HT) to the stabilization of the AcrB_EC_-ligand complex, which is higher for chlorpromazine (and comparable to that seen for ethidium bromide) than for amitriptyline (Table 6). The contributions to the estimated binding affinities of chlorpromazine and amitriptyline from residues within the DP_T_ became similar in *S.* Typhimurium, although the interaction with the HT remained tighter for the former compound. Note that although we reported the (solvation) binding free energies of the various compounds in Table 6, these should be considered approximate (qualitative) estimates of the binding affinity. This is due to the well-known limitations of the molecular mechanics combined with generalized Born and surface area continuum solvation (MM/GBSA) method (62) and to the inability to obtain converged values of the conformational entropy of binding, which when combined with the solvation free energies, should provide a more realistic estimate of the true affinities (see reference 20). This is the reason why we focused, as described above, on the structural analysis of the binding poses as well as the comparison with experimental structures of E. coli AcrB in complex with known inhibitors.

**TABLE 5:** Number of atom clashes between atoms of chlorpromazine and amitriptyline and those of substrates norfloxacin and ethidium bromide and those of the inhibitor MBX3132 bound to AcrB_EC_ (PDB ID 5ENQ)^*a*^

Compound^*b*^	No. of atomic clashes		
	Norfloxacin	Ethidium bromide	MBX3132
CPZ_EC_	4	11	14
AMI_EC_	3	0	3
CPZ_ST_	6	3	15^*c*^
AMI_ST_	5	4	None^*c*^

a The calculation was performed on the representative structure of the most populated cluster extracted from each MD trajectory (in the case of amitriptyline and chlorpromazine, we selected the trajectories associated with the more negative binding free energies among those displaying a stable position of the ligand in the last 50 ns of the production run). In addition, we used the crystal structure of E. coli AcrB in which the inhibitor MBX3132 has been cocrystallized (PDB ID 5ENQ). To evaluate the number of clashes, these structures were superimposed, and the number of heavy atoms of amitriptyline/chlorpromazine that overlap the other compounds was recorded.

b CPZ, chlorpromazine; AMI, amitriptyline.

c Under the hypothesis that MBX3132 binds to AcrB_ST_ similarly to the mode found in the X-ray structure 5ENQ of AcrB_EC_.

**TABLE 6:** Binding free energies to the DP_T_ of AcrB_EC_ and AcrB_ST_, calculated with the MM/GBSA approach^*a*^

Organism	Compound	Δ*G*_b_ (kcal/mol)	DP	HT
E. coli	CPZ	−31.9 (4.0)	−13.9	−8.9
	AMI	−25.6 (3.4)	−9.1	−6.6
	NOR	−36.4 (5.2)	−10.0	−6.5
	EtBr	−43.5 (2.9)	−14.6	−10.9
	MBX3132	−51.7	−19.6	−13.4
*S*. Typhimurium	CPZ	−25.7 (3.1)	−10.3	−8.4
	AMI	−27.7 (3.2)	−10.8	−5.7
	NOR	−29.8 (3.3)	−12.7	−10.1
	EtBr	−34.8 (2.9)	−14.0	−8.7

a The absolute values of Δ*G*_b_ are reported with standard errors in parentheses together with the contribution to stabilization of the complexes from residues lining the DP and the HT. For comparison, data for MBX3132 bound to AcrB_EC_ are also reported (22). CPZ, chlorpromazine; AMI, amitriptyline; EtBr, ethidium bromide.

10.1128/mBio.00465-20.2 FIG S2 Representative conformations of the most stable binding modes of norfloxacin and ethidium bromide within the DP_T_ of AcrB_EC_ and AcrB_ST_, as obtained from all-atom MD simulations of the periplasmic portion of the transporter in explicit solvent (see Materials and Methods for details and Fig. S3). The protein is represented as grey ribbons, while the substrates are shown as sticks colored by element (C, N, and O in dark yellow, blue, and red, respectively). Side chains of residues within 3.5 Å from the drug are represented as sticks colored by residue type (hydrophobic, polar, acid, and basic residues in purple, lime, red, and blue, respectively) and labeled. Side chains of residues belonging the DP_T_ and the phenylalanines defining the HT (see Table S1) for the definition of different regions) are also shown as transparent surfaces, colored in red and magenta, respectively. NOR, norfloxacin; EtBr, ethidium bromide. Download FIG S2, PDF file, 0.2 MB. Copyright © 2020 Grimsey et al. 2020 Grimsey et al. This content is distributed under the terms of the Creative Commons Attribution 4.0 International license.

10.1128/mBio.00465-20.3 FIG S3 Plots of the RMSDs (Å) of the protein (Cα) and of the ligand (nonhydrogenous atoms) with respect to the initial conformation of the MD simulation and of the ligand with respect to the last structure sampled during the production MD trajectory. The RMSD of the ligand was calculated after structural alignment of the monomer T of AcrB. We only report the profiles for the most stable MD simulation among the three performed for each system (see Table S2). Download FIG S3, PDF file, 1.0 MB. Copyright © 2020 Grimsey et al. 2020 Grimsey et al. This content is distributed under the terms of the Creative Commons Attribution 4.0 International license.

10.1128/mBio.00465-20.8 TABLE S3 Residues of AcrB interacting with the polar tail of amitriptyline and chlorpromazine along the MD trajectories. CPZ, chlorpromazine; AMI, amitriptyline. Download Table S3, PDF file, 0.09 MB. Copyright © 2020 Grimsey et al. 2020 Grimsey et al. This content is distributed under the terms of the Creative Commons Attribution 4.0 International license.

Chlorpromazine and amitriptyline featured a significant (limited) steric clash with ethidium bromide within the DP_T_ in AcrB_EC_ (AcrB_ST_) and AcrB_ST_ (AcrB_EC_), respectively (Fig. 8 and Table 5). The overlap between binding poses was greatly reduced for norfloxacin, which is found on top of chlorpromazine in both AcrB_EC_ and AcrB_ST_, above amitriptyline in AcrB_EC_, and below amitriptyline in AcrB_ST_.

Recently, Zwama et al. discovered a new putative entry site in AcrB of E. coli, named channel 3 (CH3) and lined by residues A33, T37, A100, G296, and N298 (63). Inspection of our blind docking results (Fig. S1) revealed that in both E. coli and *S.* Typhimurium, chlorpromazine, but not amitriptyline, binds just beneath the CH3 channel. The binding poses in this region would clash with several poses found for norfloxacin and ethidium bromide in the same region of AcrB (see Fig. S4). Notably, for both substrates, the numbers of poses behind this entry gate were greater in AcrB_EC_ than in AcrB_ST_ for corresponding monomers (L or T), while the numbers of chlorpromazine or amitriptyline poses in the proximity of CH3 were fairly similar.

10.1128/mBio.00465-20.4 FIG S4 Overlap between docking poses of norfloxacin and ethidium bromide with chlorpromazine beneath the CH3 entry gates of monomers L and T in AcrB_EC_ and AcrB_ST_. The conformations of the substrate and the inhibitor are shown as sticks, with C atoms in lime and cyan colors, respectively. Sidechains of residues lining the CH3 entry (with the addition of residue 296, possibly involved in the recognition of carboxylated compounds [1]) are shown as orange semitransparent surfaces. (A) Overlap between the docking poses of norfloxacin and chlorpromazine. (B) Overlap between the docking poses of ethidium bromide and chlorpromazine. CPZ, chlorpromazine; AMI, amitriptyline; EthBr, ethidium bromide; NOR, norfloxacin. Download FIG S4, PDF file, 0.4 MB. Copyright © 2020 Grimsey et al. 2020 Grimsey et al. This content is distributed under the terms of the Creative Commons Attribution 4.0 International license.

## DISCUSSION

Chlorpromazine and amitriptyline have been identified as potential efflux inhibitors (30). However, their mode of efflux inhibition is poorly understood, and the nature of their interaction with multidrug efflux proteins is unknown.

To facilitate the identification of the primary mode of action of chlorpromazine, chlorpromazine-resistant mutants were selected. The difficulties in selecting resistant mutants and the low mutation rates observed when mutants were generated suggest that resistance to chlorpromazine is a rare event. Chlorpromazine resistance was revealed to occur via mutations in *ramR* and *marR* of *S.* Typhimurium and E. coli, respectively. The hypothesis that these mutations may occur in the binding site of chlorpromazine was not supported. This was based on the roles of RamR and MarR; in their resting state, they bind to the promoter regions of the transcriptional activators, *ramA* and *marA*, respectively, thereby preventing their overexpression. Upon ligand binding, the conformations of RamR and MarR are altered and the proteins are unable to bind DNA. Subsequently, *ramA* and *marA* are derepressed, and overproduction of RamA and MarA occurs with accompanying overexpression of *acrAB-tolC.* Given that ligand binding is essential for derepression of the AcrAB-TolC regulatory system, it seems unlikely that these mutations simply prevent the binding of chlorpromazine. Instead, it is proposed that chlorpromazine is a substrate of AcrB and that mutations in RamR and MarR provide resistance to chlorpromazine by overexpressing *ramA* and *marA*, respectively. This consequently increases the efflux of this compound by AcrAB-TolC.

Upregulation of *acrA*, *acrB*, *tolC*, and *ramA* has been shown to occur upon exposure to certain AcrAB-TolC substrates in order to promote extrusion of a given antibiotic (39, 64). The chlorpromazine-induced upregulation of these AcrAB-TolC efflux genes is further evidence to suggest that chlorpromazine may itself be a substrate of the AcrAB-TolC efflux pump. This is supported by previous observations that hypersusceptibility to chlorpromazine occurs in strains with deletions in efflux pump genes (*acrB*, *acrD*, *acrF*, and *tolC*) or regulatory genes (*marA* and *ramA*) (30, 65). In the presence of chlorpromazine, upregulation of the transcriptional activator *ramA* and of the repressor *ramR* was also observed. Found directly upstream of *ramA*, *ramR* encodes a TetR transcriptional repressor, RamR, which binds to the promoter region of *ramA*, preventing overexpression of AcrAB-TolC; in the presence of some AcrB substrates, this binding is abolished (18, 66). Upregulation of this transcriptional repressor suggests a negative feedback loop to reduce the increased expression of AcrAB-TolC. With 95.92% of gene expression changes significantly altered in the same direction, the RNA-seq data obtained after exposure to chlorpromazine indicates that this drug behaves in a very similar manner to PaβN. Considered to be a competitive inhibitor, PaβN is a substrate of AcrB, binding to the hydrophobic trap (41). The binding of this compound to AcrB alters the efflux of other substrates by interfering with their binding to the transporter, thereby allowing intracellular accumulation that is essential for the antibacterial activity of antibiotic agents (54).

A mutation selection experiment was designed in which resistance to chlorpromazine, amitriptyline, minocycline, and spectinomycin was selected in *S.* Typhimurium with a preexisting mutation conferring a D408A substitution within the proton relay network of AcrB (17). This mutation renders AcrAB-TolC nonfunctional without affecting *in vitro* bacterial growth. The hypothesis is that exposure to an AcrB substrate would apply pressure that would select for “mutants” with a wild-type sequence (revertants) and thus a functional AcrAB-TolC efflux pump. Exposure to chlorpromazine and amitriptyline resulted in the reversion of 100% of *S.* Typhimurium D408A mutants, suggesting that these compounds are both substrates of AcrB. The observation that exposure to minocycline or ethidium bromide resulted in the reversion of 2% or 3% of *S.* Typhimurium D408A mutants, respectively, suggests that this may be a feature shared with well-characterized AcrB substrates. The evidence that exposure to spectinomycin, a non-AcrB substrate, does not induce reversion further supports that this genotypic change has the potential to identify AcrB substrates. However, it is important to note that the low reversion rate of the minocycline and ethidium bromide mutant limits its usefulness to identify all AcrB substrates. The discrepancy between the reversion rate for chlorpromazine and amitriptyline versus minocycline and ethidium bromide may be due to the AcrB-specific inhibitory properties of chlorpromazine and amitriptyline, whereas there is evidence to suggest minocycline and ethidium bromide can be exported via other pumps (67, 68). Molecular simulations show that chlorpromazine and amitriptyline are able to bind to AcrB, suggesting that they may interfere with binding by other substrates. Therefore, they may also exert a higher degree of selective pressure that drives for the reversion of AcrB to its functional wild-type state. Given this is a feature that appears to be selective for compounds with efflux inhibitory properties, there is the potential for this assay to be used to identify competitive inhibitors of AcrB.

To confirm whether chlorpromazine and amitriptyline are efflux inhibitors, we performed synergy assays and efflux and accumulation assays on *S.* Typhimurium and E. coli. Both chlorpromazine and amitriptyline have some intrinsic antibacterial activity, but at concentrations higher than those clinically achievable or desirable. Our data show that while synergy was observed between amitriptyline or chlorpromazine and certain AcrAB-TolC substrates for *S.* Typhimurium, no synergy was observed using any combination against E. coli in checkerboard assays. However, given that checkerboard assays rely on the use of doubling dilutions, for drugs such as amitriptyline and chlorpromazine with activity at high concentrations, the effective concentrations may fall between two dilutions, and small differences in susceptibility will not be observed. Therefore, we performed disk and well diffusion assays in which differences in susceptibility are more readily detected. The results indicate that both chlorpromazine and amitriptyline are able to potentiate the activities of AcrB substrates, including ethidium bromide and norfloxacin, against *S.* Typhimurium and E. coli.

**FIG 9:**
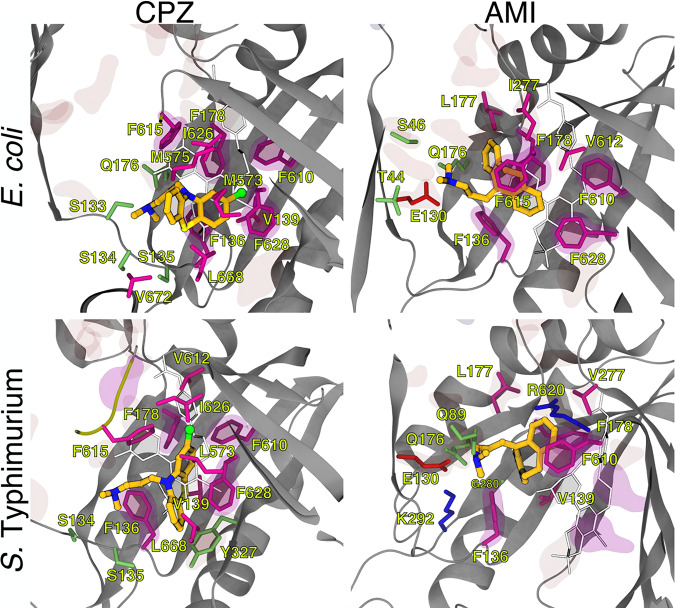
Comparison between representative conformations of the most stable binding modes of chlorpromazine and amitriptyline. Drugs are shown within the DP_T_ of AcrB_EC_ and AcrB_ST_ and the experimental structure (shown as CPK colored by element) of the pyranopyrimidine inhibitor MBX3132 in AcrB_EC_ (shown as white sticks). See the legend for Fig. 8 for details.

Furthermore, our results showed that chlorpromazine and amitriptyline acted as efflux inhibitors; both compounds increased the intracellular accumulation of ethidium bromide and norfloxacin for all strains tested. In addition, we attempted to determine whether chlorpromazine could compete for efflux with steady-state levels of the AcrAB-TolC substrate norfloxacin. Unfortunately, the degree to which chlorpromazine intrinsically fluoresces was insufficient to allow these experiments to be performed with the available equipment. *In silico* investigations were performed to shed light on the molecular determinants behind the inhibitory action of these compounds. The computational data confirm that both can bind to the distal pocket of AcrB, interacting fully or partly with the hydrophobic trap shown to be a preferred binding site for efflux inhibitors (20, 22, 41, 59). In E. coli, chlorpromazine features significant overlap with the experimental binding pose of the potent efflux inhibitor MBX3132 (22) (Fig. 9 and Table 5). In the hypothesis that MBX3132 binds to AcrB_ST_ in a similar way as in AcrB_EC_, large overlap would also arise in *S.* Typhimurium.

On the basis of the similarities with the binding mode of MBX3132, a possible mechanism of inhibition for chlorpromazine could be competitive binding with substrates within the DP_T_. Alternatively, as already suggested for doxorubicin in the F610A variant of AcrB (51) and for other substrates or efflux inhibitors (20, 22, 54, 59, 69, 70), binding of chlorpromazine can retard or hinder some functional conformational changes occurring in AcrB during substrate transport. Note that the latter hypothesis does not preclude simultaneous binding of the inhibitor and of the substrate to different monomers of AcrB, whose existence in conformational states such as LLT, LTT, or TTT, different from the resting (LLL) and fully asymmetric (LTO) ones, was confirmed by experiments on the transporter alone and on the fully assembled efflux pump (17, 22).

In contrast to chlorpromazine, amitriptyline binds upwards with respect to MBX3132, showing small or no overlap with this inhibitor in AcrB_EC_ or AcrB_ST_, respectively (Fig. 9 and Table 5). The tighter interaction of chlorpromazine with the hydrophobic trap could be due to the additional chlorine atom in this compound, establishing tight C-Cl···π interactions (71) with the aromatic rings of two or even three phenylalanine residues located in this region. Consistent with these findings, the overall lower inhibitory effect of amitriptyline than of chlorpromazine (the only exception being the impact on accumulation of ethidium bromide in *S.* Typhimurium, which was comparable for the two compounds) (Fig. 6 and 7) could be ascribed to its weaker interaction with hydrophobic residues within the pocket, particularly with the hydrophobic trap (Table 6). This should result in a weaker binding competition with substrates and/or a reduced impairment of the concerted protein motions associated with the functional rotation of AcrB.

In addition, our *in silico* findings suggest that chlorpromazine, but not amitriptyline, could interfere with the uptake of norfloxacin and ethidium bromide at the CH3 entry gate recently discovered in AcrB_EC_ (63) (see Fig. S3 and S4 in the supplemental material). While CH3 was suggested to be the preferred binding site for the class of planar, aromatic, and cationic compounds, it should be noted that (i) both chlorpromazine and amitriptyline are cationic but not planar compounds; however, the phenothiazine ring of chlorpromazine confers the molecular core of this molecule a flatter conformation than that assumed in amitriptyline (see Fig. S5). (ii) Despite that ethidium bromide, but not norfloxacin, belongs to the class of compounds for which the CH3 entry was suggested as the preferred binding site, triple (A33W/T37W/N298W) and quadruple (A33W/T37W/A100W/N298W) mutants with amino acid substitutions in this channel resulted in 3- and 2-fold changes in the MICs of ethidium bromide and norfloxacin, respectively (see Table 1 in reference 63). We speculate that the larger increase in the accumulation of norfloxacin upon coadministration of chlorpromazine rather than amitriptyline could be also due, at least in part, to competition for binding at the CH3 entrance gate. Overall, our findings allow a plausible and consistent rationale to be proposed for the different inhibitory potency of chlorpromazine and amitriptyline in *S.* Typhimurium and E. coli.

10.1128/mBio.00465-20.5 FIG S5 Comparison between equilibrium three-dimensional (3D) structures of chlorpromazine and amitriptyline. The rings building the molecular core of the two compounds are shown in CPK representation, with C atoms colored mauve and orange for chlorpromazine and amitriptyline, respectively. The tails are shown as lines colored with the same scheme. Download FIG S5, PDF file, 0.03 MB. Copyright © 2020 Grimsey et al. 2020 Grimsey et al. This content is distributed under the terms of the Creative Commons Attribution 4.0 International license.

In summary, experimental data from mutant selection experiments suggested that chlorpromazine and amitriptyline are substrates of AcrB. Corroborating this, further *in silico* work demonstrated that both compounds are able to bind the distal pocket, partly occupying the hydrophobic trap. This observation that chlorpromazine and amitriptyline are AcrB substrates may explain the efflux inhibitory properties of these compounds. We propose that both chlorpromazine and amitriptyline are substrates of AcrAB-TolC capable of binding to residues of AcrB that are important for substrate recognition and/or transport and therefore may either competitively inhibit efflux of other substrates by AcrB or act by impairing the functional rotation of AcrB.

## MATERIALS AND METHODS

### Strains and media used.

Strains used in this study are listed in Table 7. Bacterial strains were grown overnight at 37°C in Lennox broth (Sigma-Aldrich, UK) or Iso-Sensitest broth (Oxoid, UK). All chemicals and antibiotics were supplied by Sigma-Aldrich, UK. Antimicrobial disks were supplied by Oxoid, UK.

**TABLE 7:** Bacterial species used throughout this project

Strain	Genotype or phenotype	Source or reference
*Salmonella* Typhimurium		
SL1344	Wild type	94
SL1344 *ramR*::*aph*	*ramR*::*aph S.* Typhimurium SL1344 overexpressing *acrAB-tolC*	95
SL1344 AcrB (D408A)	*S.* Typhimurium containing a substitution (D408A) within AcrB rendering the pump nonfunctional	17
SL1344 *ramR* L158P	S. Typhimurium containing a mutation (L158P) within *ramR*	This study
Acinetobacter baumannii AB211	Post-tigecycline therapy isolate overexpresses *adeABC*	96
Pseudomonas aeruginosa K1454	*nalC* mutant of PAO1 overexpressing MexAB-OprM	97
Escherichia coli		
BW25113 *marR*::*aph*	*marR*::*aph* E. coli BW25113 overexpressing *acrAB-tolC*	98
MG1655	Wild type	99
MG1655 *marR* 141_142del	E. coli with a deletion within *marR*	This study
MG1655 *marR* 104delC	E. coli with a deletion within *marR*	This study

### Mutant selection.

Chlorpromazine-resistant *S.* Typhimurium SL1344 and E. coli MG1655 mutants were selected on chlorpromazine-containing Lennox agar at concentrations of 150 μg/ml and 170 μg/ml, respectively. To select for *S.* Typhimurium D408A revertants, chlorpromazine, amitriptyline, minocycline, ethidium bromide, and spectinomycin were incorporated into Lennox agar at concentrations higher than MIC values (60 μg/ml, 110 μg/ml, 0.5 μg/ml, 64 μg/ml, and 128 μg/ml, respectively). The plates were then inoculated with 1 × 10^9^ CFU/ml and were incubated aerobically at 37°C for up to 7 days or until the appearance of colonies, which were counted. The mutation rate was calculated using the mean sum of squares (MSS)-maximum likelihood method (72). To ensure confidence in the data from the experiments, agar plates were inoculated with 100 parallel cultures (divided equally from three biological replicates).

### Susceptibility to antibiotics and synergy testing.

The MIC was determined according the European Committee on Antimicrobial Susceptibility Testing (EUCAST) methodology using the doubling dilution method on agar (73). As recommended by EUCAST, E. coli ATCC 25922 was used as the quality control. The MIC of each agent was recorded as the lowest concentration of compound that prevented visible growth after 16 h and was taken as the mean from three independent biological repeats.

To determine whether chlorpromazine and amitriptyline are able to potentiate the activity of AcrB substrates, the MICs of chloramphenicol, ciprofloxacin, nalidixic acid, tetracycline, and ethidium bromide were determined in combination with doubling dilution concentrations of chlorpromazine and amitriptyline. The MIC of each agent was determined as the lowest concentration of compound that prevented visible growth after 16 h and was taken as the mean from three independent biological replicates. The full method is detailed in the supplemental material.

The ability of chlorpromazine and amitriptyline to potentiate AcrB substrates was also determined using a disk diffusion assay. Iso-Sensitest agar plates containing 55 μg/ml or 110 μg/ml of amitriptyline, 25 μg/ml or 50 μg/ml of chlorpromazine, or 50 μg/ml of PaβN were prepared to 25 ml in 90-mm petri dishes. The surface of the agar was dried thoroughly before use by incubating for 10 min at 60°C. Several morphologically similar colonies of *S.* Typhimurium SL1344 *ramR*::*aph* or E. coli BW25113 *marR*::*aph* (both overexpress AcrAB-TolC) were suspended in a saline solution to a McFarland density of 0.5. The plates were inoculated by swabbing the suspension over the entire surface of the agar. Antimicrobial disks containing chloramphenicol (30 μg), tetracycline (30 μg), nalidixic acid (30 μg), or ciprofloxacin (5 μg) were applied to the surface of the agar and the plates incubated at 37°C for 16 h. The size of the zone of inhibition was measured using a ProtoCOL 3 automated colony and zone sizing system (Don Whitley Scientific, UK). Three independent biological replicates, each with three technical replicates, were used.

A well diffusion assay was also used to determine the ability of chlorpromazine and amitriptyline to potentiate ethidium bromide and norfloxacin. Iso-Sensitest agar plates containing 55 μg/ml or 110 μg/ml of amitriptyline or 25 μg/ml or 50 μg/ml of chlorpromazine were prepared to 25 ml in 90-mm petri dishes. The surface of the agar was dried thoroughly before use by incubating for 10 min at 60°C. The surface of the agar was inoculated by spreading 50 μl of overnight cultures of *S.* Typhimurium SL1344 *ramR*::*aph* or E. coli BW25113 *marR*::*aph* across the surface of the agar. A hole of 8 mm was punched using a sterile metal borer, and 50 μl of ethidium bromide or norfloxacin was added to the wells at final concentrations of 750 μg/ml or 5 μg/ml, respectively. The agar plates were incubated at 37°C for 16 h. The size of the zone of inhibition was measured using a ProtoCOL 3 automated colony and zone sizing system (Don Whitley Scientific, UK). Three independent biological replicates, each with three technical replicates, were used.

### Whole-genome sequencing.

Mutants deemed “resistant” were whole-genome sequenced using the paired-end Illumina HiSeq 4000 platform (BGI Tech Solutions, Hong Kong). For clones where a single phenotype was observed, one mutant was sequenced. The Illumina sequencing was assembled *de novo* using VelvetOptimser 1.1.0 within the Microbial Genomic Virtual Lab (GVL) Galaxy version 4.1.0 (74). The genomes of the parental strains were annotated by Rapid Annotation using Subsystem Technology (RAST) (75). To identify single nucleotide polymorphisms (SNPs) and indels, snippy was used to map the assembled genomes of the mutant strains against the respective parental strain.

### Allele-specific quantitative PCR.

Allelic specific PCR was used to identify the presence or absence of 1223G conferring the D408A substitution within *S.* Typhimurium SL1344. This method allows the detection of single SNPs by the use of two allele-specific primers that only amplify their complementary allele: one specific for the wild-type allele (408D) and one specific for the mutant allele (408A). Although this method is typically performed with primers differing in their terminal 3′ nucleotides, because the energy binding cost of the mutant allele primer is insufficient to prevent binding to the wild-type sequence, an additional mismatch was incorporated at the penultimate nucleotide in order to destabilize the base pairing between the primers and the corresponding nontarget template. Primer sequences were as follows: *S.* Typhimurium 408A forward, CATCGGCTTGCTGGTGGATAC; *S.* Typhimurium wild-type 408D forward, CATCGGCTTGCTGGTGGATGA; *S.* Typhimurium *acrB* reverse, GATCGGCCCAGTCTTTCAACG.

Each mutant candidate was screened for both the 408D allele (wild type) and the 408A allele (mutant) using real-time quantitative PCR. Each mutant was cultured on lysogeny broth. Bacteria from each colony were transferred into 50 μl of molecular-grade water in a 96-well PCR plate, and the cells were lysed by heating at 99°C for 10 min. The PCR mixture was made to a final volume of 20 μl containing 1 μl of lysate and iQ Sybr green supermix (Bio-Rad), prepared according to the manufacturer’s instructions with an appropriate combination of primers. The quantitative PCR was performed on a Bio-Rad C1000 thermocycler using the following cycling conditions: 95°C (3 min), 95°C (15 min, 30 cycles), and 72°C (40 min, 30 cycles). A melting curve was generated using a temperature range from 50°C to 95°C with increments of 0.5°C every 5 s.

### RNA extraction, sequencing, and bioinformatics analysis.

*S.* Typhimurium SL1344 was grown to an optical density at 600 nm (OD_600_) of 0.6 in MOPS (morpholinepropanesulfonic acid) minimal medium supplemented with 2.6 mM l-histidine. The cells were then exposed to chlorpromazine (50 μg/ml) for 2 h. The RNA was extracted using an SV total isolation system (Promega, USA), and the concentrations of RNA and DNA were quantified using Qubit fluorimetric quantification. DNase treatment with the Turbo DNA-free kit (Thermo Fisher Scientific, UK) was performed, if required. The purity of the RNA samples was determined using a NanoDrop spectrophotometer. The samples were paired-end sequenced using an Illumina HiSeq 4000 system by BGI, Hong Kong, and the bioinformatics analysis, using FQ312003 as a reference, was undertaken as previously described (76).

### RT-PCR to determine *acrB* expression after exposure to chlorpromazine.

Three biological replicate overnight cultures of *S.* Typhimurium SL1344 were grown in MOPS minimal medium at 37°C. Following overnight growth, four starter cultures from each biological replicate were set up in MOPS minimal medium and were incubated at 37°C with shaking until an OD_600_ of approximately 0.6 to 0.8 was attained. Chlorpromazine was then added to the cultures at concentrations of 0, 50, 100, and 200 μg/ml, and incubation was continued at 37°C, with shaking, for an additional 30 min. RNA preparations were made and quantified as previously described (77). cDNA was synthesized from 2 μg of total RNA using the SuperScript III cDNA synthesis kit (Invitrogen). Quantitative RT-PCRs were set up in a Bio-Rad PCR tray using 1 μl of neat cDNA for the test gene (*acrB*) and 1 μl of a 1: 1,000 dilution of cDNA for 16S in a 25-μl reaction mixture containing 12.5 μl of iQ Sybr green supermix (Bio-Rad, UK), 1 μl of primers (500 nM), and 9.5 μl of sterile water. Primers used were as follows: 16S (*rrsH*) forward, 5′-TACCTGGTCTTGACAT-3′; 16s (*rrsH*) reverse, 5′-GACTTAACCCAACATTTC-3′; *acrB* forward, 5′-GTCCTCAAGTAGCTTCCT-3′; *acrB* reverse, 5′-GTAATCCGAAATATCCTCCTG-3′. Quantitative reverse transcription-PCR (RT-PCR) was carried out in a CFX96 real-time machine (Bio-Rad, UK) using the following protocol: 95°C for 5 min followed by 40 plate read cycles of 95°C for 30 s, 57.3°C for 30 s, and 72°C for 30 s. Data were analyzed using CFX Manager (Bio-Rad, UK) and expression ratios were calculated using the threshold cycle (ΔΔ*C_T_*) method and normalized to the expression of 16S (78).

### Efflux and accumulation assays.

The efflux of ethidium bromide was determined as previously described (79). Cells cultured and adjusted to an OD_600_ of 0.2 were supplemented with 50 μg/ml of ethidium bromide and 100 μM carbonyl cyanide-*m*-chlorophenylhydrazone (CCCP) before addition to a clear-bottomed black 96-well plate. After addition of chlorpromazine, amitriptyline, and Phe-Arg-β-naphthylamide (PaβN), the fluorescence was measured every minute, for 100 min, at 37°C in a FLUOstar Optima microplate reader (excitation and emission wavelengths 544 nm and 590 nm, respectively). The initial fluorescence was measured for 5 min before the injection of glucose to a final concentration of 25 mM.

The accumulation of norfloxacin was determined as previously described, with modifications (79). Cultures grown to an OD_600_ of 0.6 were treated with norfloxacin (10 μg/ml) and incubated for 5 min on ice; the samples were then centrifuged, washed with phosphate buffer, and resuspended in 1 ml of glycine buffer. The samples were incubated for 2 h at room temperature and centrifuged, and the fluorescence (excitation and emission wavelengths 281 nm and 440 nm, respectively) of a 1:10 dilution of the supernatant was determined.

### Homology modeling of AcrB from *Salmonella* Typhimurium.

To perform ensemble docking calculations on E. coli and *S.* Typhimurium AcrB, several homology models of the latter were built using Modeller 9.21 (80) and several E. coli AcrB X-ray structures as the templates (see Table S4 in the supplemental material). Among these, the E. coli AcrB structures labeled 5ENx were truncated at the transmembrane (TM) region, and the protein assumed the LLT conformation. Therefore, we first generated their full structural models in the LTO conformation via homology modeling with multiple templates, as follows: chains A (in the L state) and C (in the O state) of the model were built using the corresponding chains of 4DX5 as the templates; chain B of the model was built using the TM of chain B of 4DX5 and the chain C of the corresponding 5ENx structure (both in the T state) as the templates.

10.1128/mBio.00465-20.9 TABLE S4 X-ray structures of AcrB_EC_ used as structural templates to generate an ensemble of putative conformations of AcrB_ST_ by homology modelling. Download Table S4, DOCX file, 0.01 MB. Copyright © 2020 Grimsey et al. 2020 Grimsey et al. This content is distributed under the terms of the Creative Commons Attribution 4.0 International license.

10.1128/mBio.00465-20.10 METHOD S1 Method for checkerboard assay. Download Text S1, PDF file, 0.1 MB. Copyright © 2020 Grimsey et al. 2020 Grimsey et al. This content is distributed under the terms of the Creative Commons Attribution 4.0 International license.

For the modeling procedure, the amino acid sequences of the E. coli and *S.* Typhimurium AcrB transporters were first retrieved from the UniProt database (UniProt identifiers [IDs] P31224 and Q8ZRA7, respectively). The sequences were aligned using Clustal Omega (81) in order to determine the percentage of identical residues (∼95%) and verify the absence of gaps. Next, Modeller 9.21 (80) was used to build the homology models. The variable target function method was used to perform the optimization and the models with the highest MOLPDF were used for molecular docking as described below.

### Molecular docking.

Blind ensemble docking calculations were performed for amitriptyline, chlorpromazine, ethidium bromide, and norfloxacin on E. coli and *S.* Typhimurium AcrB structures using AutoDock VINA (82). As we were interested in binding poses (preferred orientation of a ligand to a protein) in the periplasmic region of AcrB, docking was performed within a rectangular search space of size 125 Å by 125 Å by 110 Å enclosing that portion of the protein, as in reference 60. The exhaustiveness parameter was set to 8,192 (∼1,000 times the default 8) in order to improve the sampling within the large box used (∼64 times the suggested volume of 30 Å by 30 Å by 30 Å). The flexibility of the receptor was considered indirectly by employing ensembles of conformations: 10 structures for each AcrB protein (E. coli and *S.* Typhimurium), while the flexibility of the ligands was considered by activating torsional angles in AutoDock VINA and using a starting structure that was optimized at the quantum-level of theory available at www.dsf.unica.it/translocation/db (83).

### Molecular dynamics simulations.

To select a tractable number of AcrB-ligand complexes on which to perform MD simulations, a cluster analysis was carried out on all the docking poses of each system, using the distance root mean square deviation (dRMSD) of the ligand as a metric to select their different orientations. The hierarchical agglomerative clustering algorithm implemented in the “cpptraj” module of the AMBER18 package (84) was used with a 3-Å dRMSD cutoff. Selected docking poses (namely, those featuring different orientations among the top ranked ones according to the AutoDock VINA scoring function) were subjected to all-atom MD simulations using the truncated model of AcrB (20, 22, 41), which includes only the periplasmic domain (residues 32 to 335 and 564 to 860 of each monomer). The AcrB-ligand complexes were inserted in a truncated octahedral box ensuring a minimum distance of 16 Å between the complex and the border of the box. The box was filled with a 0.15 M KCl aqueous solution. The topology and the initial coordinate files of the systems were created using the LEaP module of AMBER18. The AMBER force field ff14SB (85) was used to represent the protein systems; the TIP3P model was employed for water (86), and the parameters for the ions were obtained from reference 87. The parameters of amitriptyline and chlorpromazine, obtained from the GAFF force field (88) or generated using the tools of the AMBER18 package are available at www.dsf.unica.it/translocation/db (83). To improve the stability of the periplasmic region at the border with the TM domain, harmonic positional restraints (k = 1 kcal mol^−1 ^Å^−2^) were imposed on Cα atoms of residues within 5 Å from the bottom region of the structure.

Each system was first subjected to a multistep structural relaxation via a combination of steepest descent and conjugate gradient methods using the pmemd module of AMBER18, as described previously (20, 22, 41, 43). The systems were then heated from 0 to 310 K in 1.25 ns under constant pressure (set to a value of 1 atm) and with restraints on the Cα atoms found within 5 Å from the bottom of the protein. Next, a 10-ns-long MD simulation was performed to equilibrate the box dimensions, applying to the system the same restraints used for the heating procedure. This equilibration step was carried out under isotropic pressure scaling using the Berendsen barostat, whereas an Andersen thermostat (with randomization of the velocities every 500 steps) was used to maintain a constant temperature. Finally, 150-ns-long production MD simulations were performed for each system. A time step of 4 fs was used during these runs, after the protein was subjected to hydrogen-mass repartitioning (89); R-H bonds were constrained with the SHAKE algorithm. Coordinates were saved every 100 ps. The particle mesh Ewald algorithm was used to evaluate long-range electrostatic forces with a nonbonded cutoff 9 Å.

### Postprocessing of MD trajectories.

MD trajectories were analyzed using either in-house tcl and bash scripts or the cpptraj tool of AMBER18. Figures were prepared using gnuplot 5.0 (90) and VMD 1.9.3 (91).

**(i) Cluster analysis.** Clustering of the trajectories to select nonequivalent binding poses of the ligands was carried out using the average-linkage hierarchical agglomerative method implemented in cpptraj and employing a dRMSD cutoff of 2.5 Å on all the nonhydrogenous atoms of the ligand.

**(ii) Binding free energy calculations.** The MM/GBSA approach (62) implemented in AMBER18 was used to calculate the solvation free energies following the same protocol used in previous studies (20, 22, 41, 54, 92). This approach provides an intrinsically simple method for decomposing the free energy of binding into contributions from single atoms and residues (93). The solute conformational entropy contribution (TΔS_conf_) was not evaluated (62). Calculations were performed on 50 different conformations of each complex, which were extracted from the most populated conformational cluster (representing the most sampled conformation of the complex along the production trajectories).

**(iii) Ligand flexibilities.** The root mean square fluctuations (RMSFs) of the ligands were calculated using cpptraj after structural alignment of each trajectory.

### Data availability.

The paired-end RNA sequencing data are available in ArrayExpress (accession no. E-MTAB-8190).
