# Supplementary figures and images for: Chlorpromazine and Amitriptyline Are Substrates and Inhibitors of the AcrB Multidrug Efflux Pump
Source: mBio. 2020 Jun 2;11(3):e00465-20. doi: 10.1128/mBio.00465-20 (PMC7267879; doi:10.1128/mBio.00465-20)

Figure S1

A)

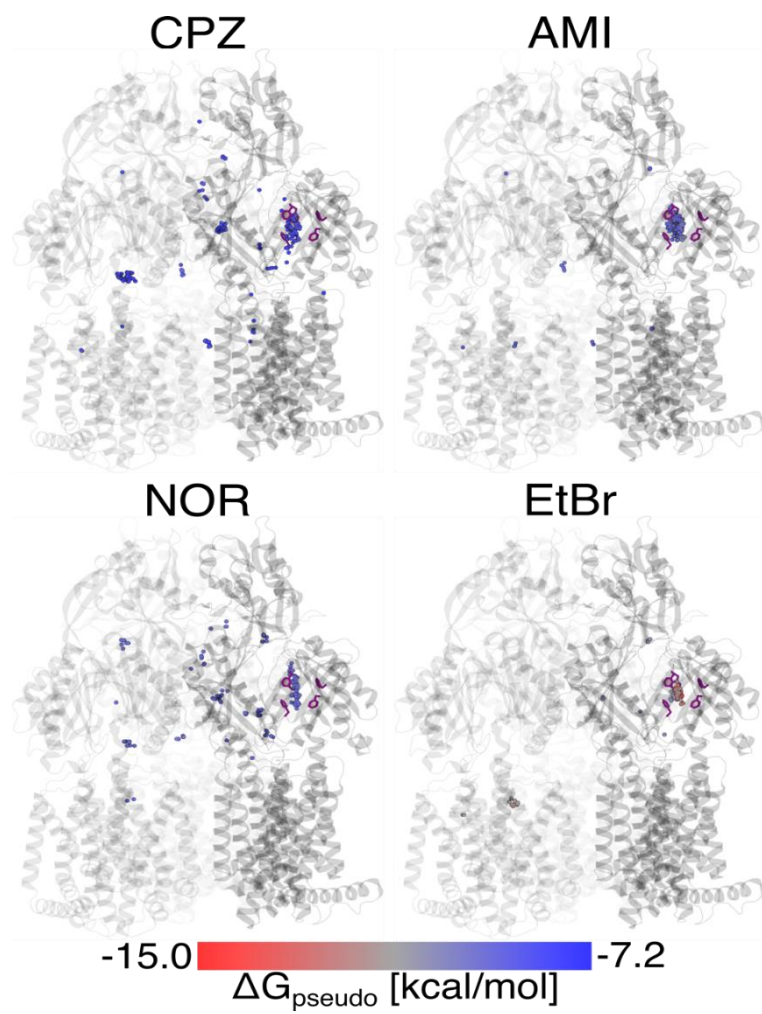

B)

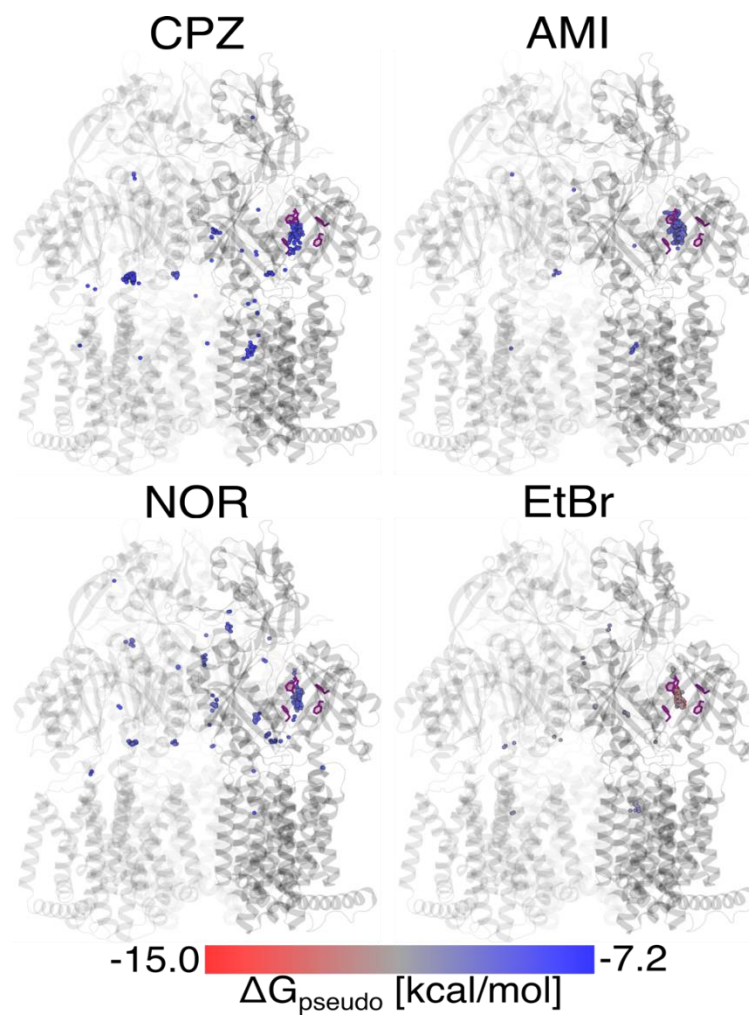

Supplement: FIG S1 [file mBio.00465-20-sf001.pdf]

Figure S2

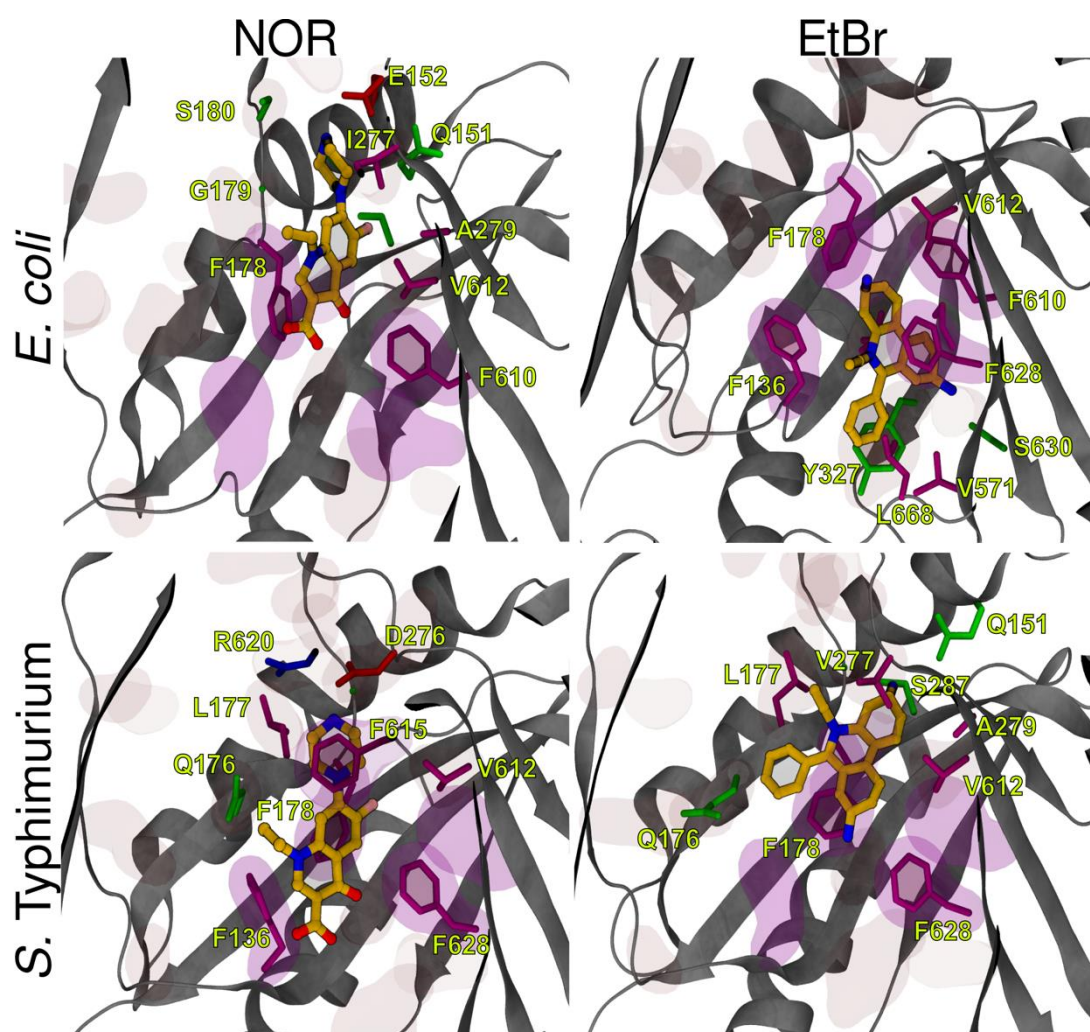

Supplement: FIG S2 [file mBio.00465-20-sf002.pdf]

**Figure S3**

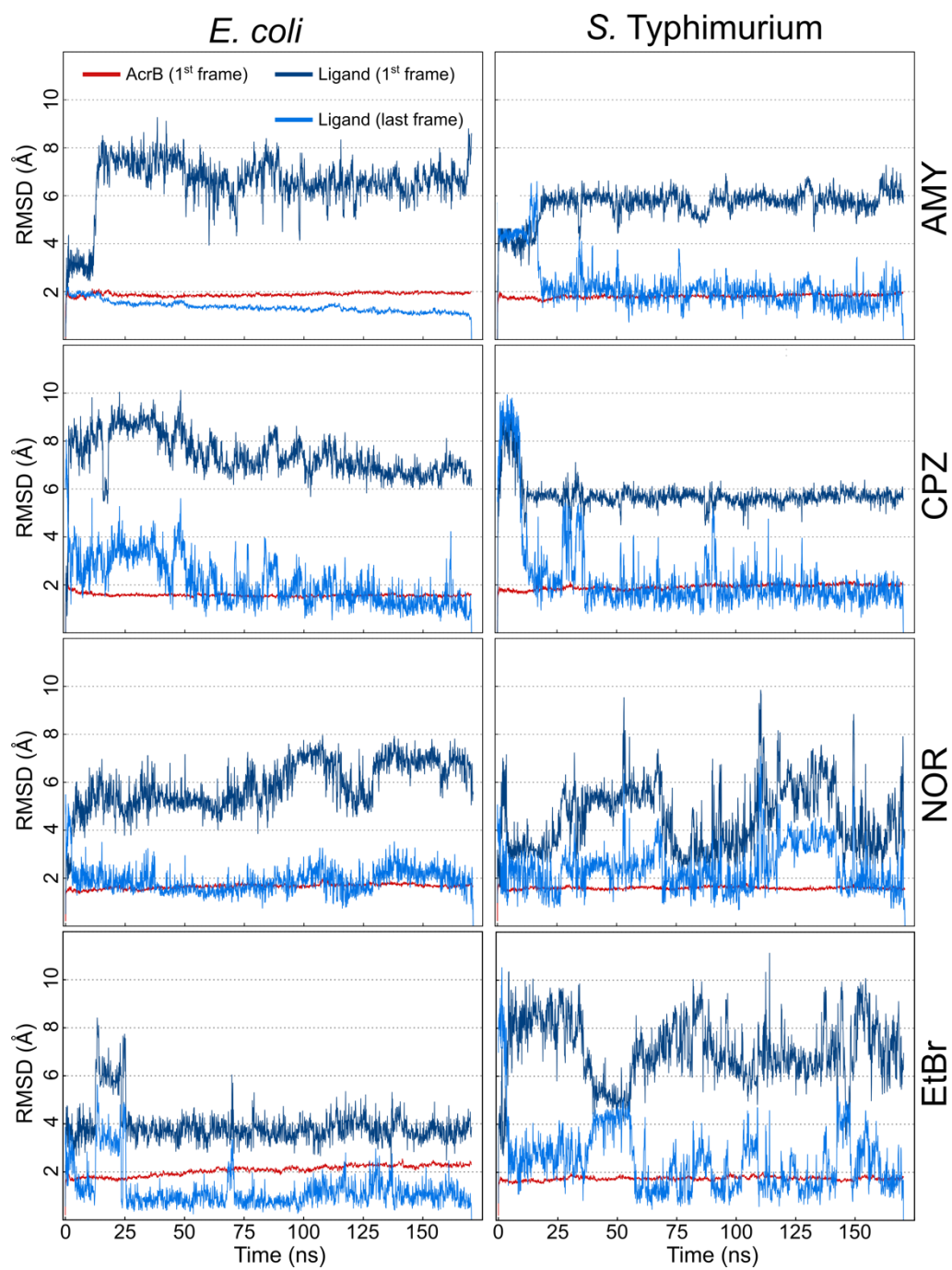

Supplement: FIG S3 [file mBio.00465-20-sf003.pdf]

Figure S4

**A) NOR and CPZ**

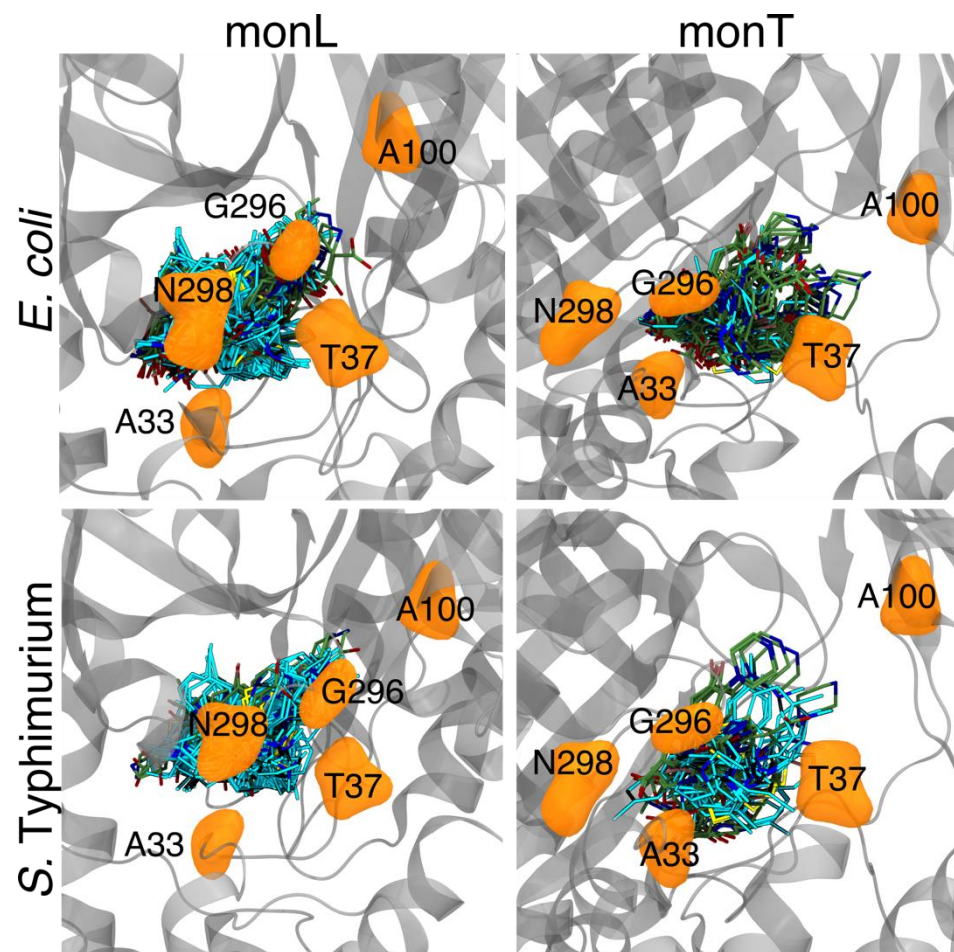

**B) EthBr and CPZ**

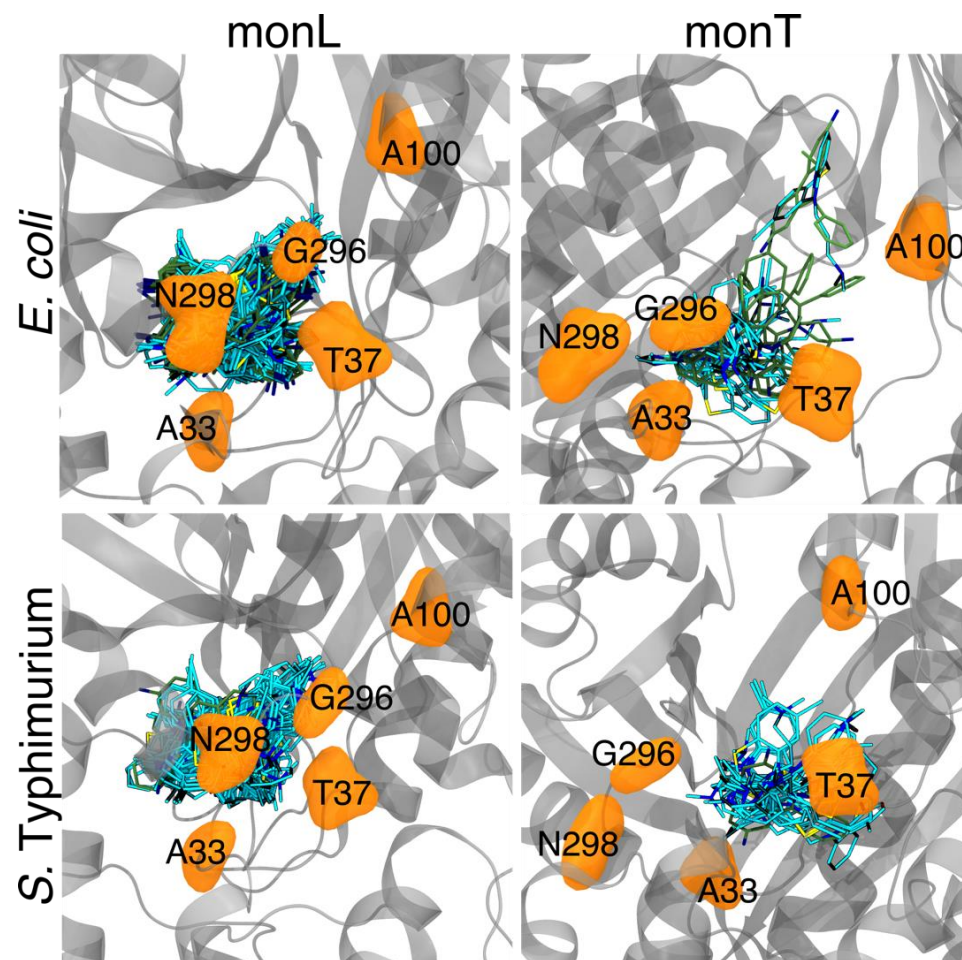

Supplement: FIG S4 [file mBio.00465-20-sf004.pdf]

**Figure S5**

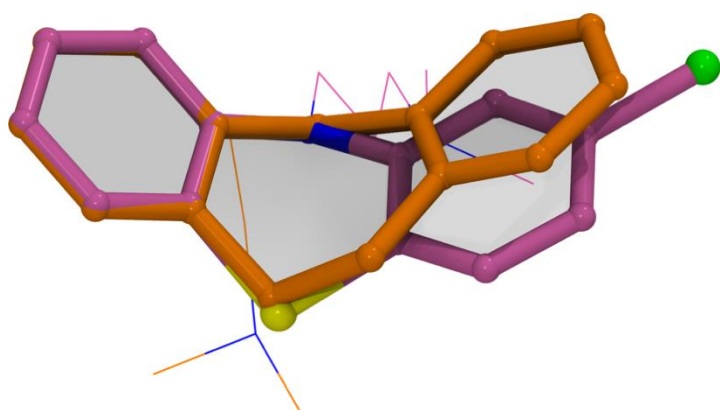

Supplement: FIG S5 [file mBio.00465-20-sf005.pdf]
