# Supplementary material for: Chlorpromazine and Amitriptyline Are Substrates and Inhibitors of the AcrB Multidrug Efflux Pump
Source: mBio. 2020 Jun 2;11(3):e00465-20. doi: 10.1128/mBio.00465-20 (PMC7267879; doi:10.1128/mBio.00465-20)
Supplement: TABLE S1 [file mBio.00465-20-st001.docx]

**Supplementary Table 1**

| Region | Lining residues |
| --- | --- |
| Distal Pocket (DP) | 46 89 128 130 134 136 139 176 177 178 179 180 273 274 276 277 327 573 610 612 615 617 620 628 |
| Hydrophobic trap (HT) | 136 178 610 615 628 |
| CH3^*^ | 33 37 100 296 298 |
| G-rich loop (G-loop) | 616 617 618 619 |

* residue 296 was not included in the definition given in (63).
