# Supplementary material for: Chlorpromazine and Amitriptyline Are Substrates and Inhibitors of the AcrB Multidrug Efflux Pump
Source: mBio. 2020 Jun 2;11(3):e00465-20. doi: 10.1128/mBio.00465-20 (PMC7267879; doi:10.1128/mBio.00465-20)
Supplement: TABLE S2 [file mBio.00465-20-st002.pdf]

**Supplementary Table 2**

| Substrate | Number of MD simulations | |
|---|---|---|
| | *E. coli* | *S.* Typh. |
| AMI | 3 | 3 |
| CPZ | 3 | 3 |
| NOR | 3 | 3 |
| EtBr | 3 | 3 |
