# Supplementary material for: Chlorpromazine and Amitriptyline Are Substrates and Inhibitors of the AcrB Multidrug Efflux Pump
Source: mBio. 2020 Jun 2;11(3):e00465-20. doi: 10.1128/mBio.00465-20 (PMC7267879; doi:10.1128/mBio.00465-20)
Supplement: TABLE S3 [file mBio.00465-20-st003.pdf]

Supplementary Table 3

AMI – $AcrB_{EC}$	E130 (69.3%), Q176 (60.0%), water-mediated interactions (4.7%)
AMI – $AcrB_{ST}$	E130 (67.1%), Q176 (64.8%), water-mediated interactions (4.6%)
CPZ – $AcrB_{EC}$	S133 (82.7%), S134 (58.9%), water-mediated interactions (17.5%)
CPZ – $AcrB_{ST}$	S134 (52.0%), S135 (44.0%), F136* (10.0%), F617* (9.8%), water-mediated interactions (10.0%)
