# Supplementary material for: Chlorpromazine and Amitriptyline Are Substrates and Inhibitors of the AcrB Multidrug Efflux Pump
Source: mBio. 2020 Jun 2;11(3):e00465-20. doi: 10.1128/mBio.00465-20 (PMC7267879; doi:10.1128/mBio.00465-20)
Supplement: TABLE S4 [file mBio.00465-20-st004.docx]

**Supplementary Table 4**

| PDB ID | Resolution (Å) | Co-crystallized compound |
| --- | --- | --- |
| 2J8S  (71) | 2.5 | - |
| 4DX5  (12) | 1.9 | Minocycline |
| 4DX7  (12) | 2.3 | Doxorubicin |
| 5NC5  (54) | 3.2 | Puromycin |
| 5EN5* | 2.3 | - |
| 5ENO* | 2.2 | MBX2319 (inhibitor) |
| 5ENP* | 1.9 | MBX2931 (inhibitor) |
| 5ENQ* | 1.8 | MBX3132 (inhibitor) |
| 5ENR* | 2.3 | MBX3135 (inhibitor) |
| 5ENS* | 2.8 | Rhodamine-6G |

*Truncated structures containing only the periplasmic domain of the protein (22).
