# Supplementary material for: Chlorpromazine and Amitriptyline Are Substrates and Inhibitors of the AcrB Multidrug Efflux Pump
Source: mBio. 2020 Jun 2;11(3):e00465-20. doi: 10.1128/mBio.00465-20 (PMC7267879; doi:10.1128/mBio.00465-20)
Supplement: METHOD S1 [file mBio.00465-20-s0001.pdf]

Supplementary Method 1. Method for chequerboard assay

Note: A titration of the first antibiotic is made from wells 1-12 of a 96-well microtitre plate and a titration of the second drug is made from wells A-H.

Using a multi-channel pipette add 50 µl of appropriate broth to columns 2-12 of a 96-well microtitre plate. Add 50 µl of the first antibiotic (at four times the final concentration) to wells A1-H1 and A2-H2. Then using a multi-channel pipette double dilute 50 µl of antibiotic in broth from column 2 to column 11 (remove 50 µl from column 11). Do not touch column 12. Add 50 µl of the second drug (at four times the final concentration) to wells A1-A12. Using the multi-channel pipette double dilute 50 µl of drug two in broth from row A to row G (remove 50 µl from row G). Do not touch row H. This allows row H to be a control for the first antibiotic and column 12 to be a control for the second drug, well H12 will contain no antibiotic and is the control for growth of the test organism.

Dilute an overnight culture grown in iso-sensitest broth 1:1000 in iso-sensitest broth, then further dilute this suspension by 1:5. Using a multi-channel pipette add 50 µl of the final dilution to each well and mix gently. Cover the plate with a sterile lid and incubate at 37 °C for 16 hours.
